# Association Between 12 Polymorphisms of VEGF/Hypoxia/Angiogenesis Pathway Genes and Risk of Urogenital Carcinomas: A Meta-Analysis Based on Case-Control Studies

**DOI:** 10.3389/fphys.2018.00715

**Published:** 2018-06-11

**Authors:** Jin-Bo Chen, Meng Zhang, Yu Cui, Pei-Hua Liu, Yan-Wei Qi, Chao Li, Xu Cheng, Wen-Biao Ren, Qia-Qia Li, Long-Fei Liu, Min-Feng Chen, He-Qun Chen, Xiong-Bing Zu

**Affiliations:** ^1^Department of Urology, Xiangya Hospital, Central South University, Changsha, China; ^2^Department of Urology, The First Affiliated Hospital of Anhui Medical University, Hefei, China; ^3^Beijing Genomics Institute, Shenzhen, China

**Keywords:** VEGF/hypoxia/angiogenesis, polymorphism, urogenital carcinomas, susceptibility, meta-analysis

## Abstract

**Objective:** Previous studies indicated potential associations between polymorphisms in genes of VEGF/hypoxia/angiogenesis pathway and risk of urogenital carcinomas However, the results were controversial and inconclusive. Here, we conducted an in-depth meta-analysis to investigate the precise associations between polymorphisms in VEGF/hypoxia/angiogenesis related genes and risk of urogenital carcinomas.

**Methods:** We searched PubMed, Web of Science, EMBASE, and Cochrane Library to identify all eligible publications. Pooled odds ratios (ORs) corresponding with the 95% confidence intervals (CIs) were calculated to evaluate their associations. Subgroup analysis was conducted to further ascertain such relationship and investigate sources of heterogeneity.

**Results:** In the end, a total of 96 case-control studies fulfilled the inclusion criteria were enrolled for 12 polymorphisms in 4 VEGF/hypoxia/angiogenesis related genes. The pooled results showed eNOS-rs2070744 polymorphism conferred a significantly increased overall risk of urogenital carcinomas in allele, homozygote, and recessive models, respectively. In addition, eNOS-Intron 4a/b VNTR polymorphism was identified related to an increased risk of urogenital carcinomas in recessive model. And VEGF-rs699947 polymorphism was also identified an increased risk of renal cell carcinoma (RCC) in allelic, heterozygote, dominant, homozygote, and recessive models.

**Conclusion:** To conclude, eNOS-rs2070744 and eNOS-Intron 4a/b VNTR polymorphisms are risk factors for urogenital carcinomas. VEGF-rs699947 polymorphism was also identified as an increased risk factor for renal carcinoma.

## Introduction

Urogenital carcinomas, mainly including renal cell carcinoma (RCC), bladder carcinoma (BCa), and prostate carcinoma (PCa), are common malignancies among human neoplasms, with increasing morbidity worldwide in the last 2 decades (Dy et al., 2017). Based on the latest statistics in 2017, estimated incident of urogenital carcinomas accounts for about 20% of all tumors in the United States. Among them, PCa ranks the highest in man with 161,360 estimated new cases in 2017 (Siegel et al., 2017). In addition to life style and occupational exposure, numerous studies indicated genetic factors such as single nucleotide polymorphisms (SNPs) may be associated with urogenital carcinomas susceptibility (Sun et al., 2008; Stadler et al., 2010).

Angiogenesis, which is the process of new blood vessels formation from original pre-existing vessels, plays a critical role in tumor initiation and development (Nicholson and Theodorescu, 2004). The vascular endothelial growth factor (VEGF) gene is highly polymorphic and several functional SNPs in the VEGF gene alter the expression of the VEGF protein, thereby affecting tumor growth and progression (Ruggiero et al., 2011). The hypoxia-inducible factor-1 alpha (HIF1α) gene is an important transcription factor in cells which regulates cellular responses, adaption, and survival under low oxygen condition in physiological and pathological processes (Li et al., 2013). Several studies demonstrated HIF1α-rs11549465 polymorphism contributed to increase the risk of prostate cancer (Orr-Urtreger et al., 2007; Foley et al., 2009). Endothelial nitric oxide synthase (eNOS) is a central mediator of several endothelium growth stimulators, such as VEGF (Duda et al., 2004; Zhao et al., 2014). Polymorphisms of the eNOS gene plays a vital role in the angiogenesis pathway and have also been found to have functional and clinical significance in malignancies (Haque et al., 2015). HRAS (Harvey rat sarcoma viral oncogene homolog) gene has been uncovered as one of the major factors in the initiation and progression of human malignancies (Zhang et al., 2008). HRAS SNP was also noted to be associated with many cancer susceptibilities.

Clarifying of the role of VEGF/hypoxia/angiogenesis gene polymorphisms in the influence of cancer may improve our knowledge of tumor angiogenesis and benefit risk stratification, disease detection, and prognosis prediction. As mentioned above, many studies have conducted investigations to clarify the associations between these polymorphisms and the risk of urogenital carcinomas, however, these results are controversial and inconsistent. In the current study, we retrieved published data and performed a comprehensive meta-analysis to systematically investigate the association between polymorphisms in VEGF/hypoxia/angiogenesis pathway genes and the risk of urogenital carcinomas.

## Materials and methods

### Acquisition of the VEGF/hypoxia/ angiogenesis pathway gene set

The gene set of VEGF/hypoxia/angiogenesis pathway was referenced from the Kyoto Encyclopedia of Genes and Genomes (KEGG) website. The VEGF/hypoxia/angiogenesis pathway gene set could be extracted via following URL link (http://software.broadinstitute.org/gsea/msigdb/geneset_page.jsp?geneSetName=BIOCARTA_VEGF_PATHWAY&keywords=angiogenesis). The gene set was originally provided via the KEGG signaling database, and encompassed the following 29 genes: ARNT, EIF1, EIF1AX, EIF2B1, EIF2B2, EIF2B3, EIF2B4, EIF2B5, EIF2S1, EIF2S2, EIF2S3, ELAVL1, FLT1, FLT4, HIF1A, HRAS, KDR, eNOS, PIK3CA, PIK3CG, PIK3R1, PLCG1, PRKCA, PRKCB, PTK2, PXN, SHC1, VEGF, and VHL.

### Literature search strategy

We performed a literature search from PubMed, Web of Science, EMBASE, and Cochrane Central Search Library according to the PRISMA guidelines (Shamseer et al., 2015) (last research update: July 20, 2017). The search terms were as follows: (gene OR synset) AND (polymorphism OR mutation OR variation OR SNP OR genotype) AND (carcinoma OR cancer OR neoplasm OR adenocarcinoma OR tumor OR malignancy) (Supplementary Table 1). The language of enrolled studies was restricted to English. Also, we identified additional articles by screening the references of enrolled articles and reviews.

### Inclusion and exclusion criteria

Studies fitting the following inclusion criteria were enrolled: (1) articles has shown the association between polymorphisms in genes of VEGF/hypoxia/angiogenesis pathway and risk of urogenital carcinomas; (2) case-control studies; (3) publications with sufficient genotype data to assess odds ratios (ORs) and 95% confidence intervals (CIs). The exclusion criteria were as follows: (1) case-only studies without a control group, case reports, conference abstracts or reviews; (2) studies without raw data for the genotype; (3) studies with overlapping data.

### Data extraction

Two authors (JBC and MZ) individually extracted eligible data from each publication based on the inclusion and exclusion criteria. If there is a discrepancy, we reached agreement after discussing with a third author (XBZ). Information collected as follows: first author name, publication year, ethnicity, genotyping methods, source of controls including population-based (PB) or hospital-based (HB), Hardy-Weinberg equilibrium (HWE), cancer type, number of cases, and controls in the VEGF/hypoxia/angiogenesis genotypes. The Newcastle-Ottawa Scale (NOS) was used for assessing the quality of studies. High-quality study required score 7 to 9, and a score less than 7 defined as a low-quality study (Supplementary Table 2).

### Linkage disequilibrium (LD) analysis across populations

The 1,000 genomes Project database (https://www.ncbi.nlm.nih.gov/variation/tools/1000genomes/) was used to extract the LD data, which comprising the polymorphisms in VEGF, HIF-1α and eNOS evaluated in the present study. Briefly, populations enrolled in the project including JPT (Japanese in Tokyo, Japan), YRI (Yoruba in Ibadan, Nigeria), CEU (Utah residents with Northern and Western European ancestry from the CEPH collection), and CHB (Han Chinese in Beijing, China). The LD was assessed by *r*^2^ statistics in these populations using Haploview software.

### Statistical analysis

The association between polymorphisms in four VEGF/hypoxia/angiogenesis pathway genes and the urogenital carcinomas susceptibility were evaluated using summary ORs and the corresponding 95%CIs. We used allelic (B vs. A), recessive (BB vs. BA + AA), dominant (BA + BB vs. AA), homozygous (BB vs. AA), and heterozygous (BA vs. AA) models to analyze each variable (A indicate wild allele and B indicate mutated allele). Q-statistic as well as *I*^2^ were used to evaluate the heterogeneity within the studies (Higgins et al., 2003). Heterogeneity was considered significant when the *P*-value < 0.1. If there was no heterogeneity found, a fixed effect model was applied. Otherwise, the random-effect model was used to calculate pooled ORs. The significance of overall ORs was determined by Z-test. Subgroup analyses were performed based on different ethnicity, cancer type, HWE, and the source of control. Sensitivity analysis was conducted to assess the results stability by omitting one study each time. Publication bias was analyzed by Begg's funnel plot and Egger's test (Begg and Mazumdar, 1994; Egger et al., 1997). All analyses were performed using STATA 12.0 statistical software (Stata Corpotation, College Station, TX, USA).

## Results

### Main characteristics of the eligible studies

**Figure 1:**
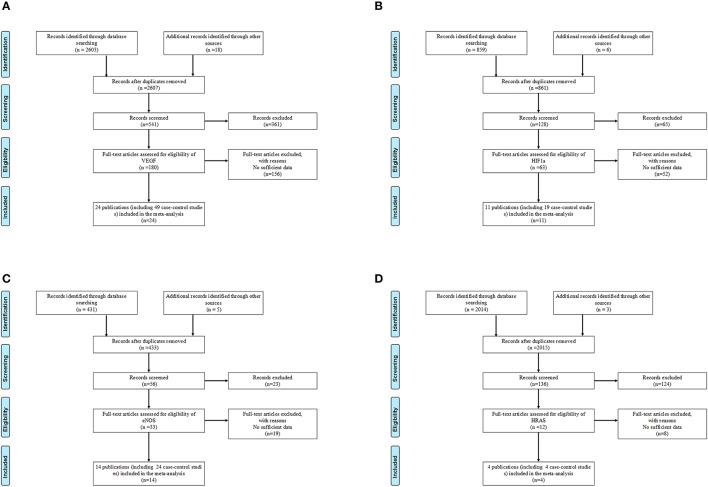
Flow chart of studies selection for VEGF/hypoxia/angiogenesis genes polymorphisms. **(A)** Flow chart of studies selection for VEGF genes polymorphisms. **(B)** Flow chart of studies selection for HIF1α genes polymorphisms. **(C)** Flow chart of studies selection for eNOS genes polymorphisms. **(D)** Flow chart of studies selection for HRAS genes polymorphisms.

According to the inclusion and exclusion criteria, only 12 polymorphisms in four genes of VEGF/hypoxia/angiogenesis pathway were identified (VEGF-rs10434, VEGF-rs1570360, VEGF-rs2010963, VEGF-rs3025039, VEGF-rs699947, VEGF-rs833061, HIF1a-rs11549465, HIF1a-rs11549467, eNOS-rs1799983, eNOS-rs2070744, eNOS-Intron 4a/b VNTR, and HRAS-rs12628). Details of the enrolled studies were listed in Table 1 (Clifford et al., 2001; Abe et al., 2002; McCarron et al., 2002; Medeiros et al., 2002; Johne et al., 2003; Lin et al., 2003; Sanyal et al., 2004; Chau et al., 2005; Kim et al., 2005; Marangoni et al., 2006; Sfar et al., 2006; Fukuda et al., 2007; Garcia-Closas et al., 2007; Li et al., 2007, 2012; Orr-Urtreger et al., 2007; Jacobs et al., 2008; Nadaoka et al., 2008; Onen et al., 2008; Foley et al., 2009; Lee et al., 2009; Morris et al., 2009; Ricketts et al., 2009; Bruyère et al., 2010; VanCleave et al., 2010; Ajaz et al., 2011; Ryk et al., 2011; Sanli et al., 2011; Amasyali et al., 2012; Henríquez-Hernández et al., 2012; Qin et al., 2012, 2014; Traczyk et al., 2012; Brankovic et al., 2013; Ianni et al., 2013; Jaiswal et al., 2013; Martinez-Fierro et al., 2013; Pandith et al., 2013; Sáenz-López et al., 2013; Safarinejad et al., 2013; Verim et al., 2013; Wang et al., 2013; Ziaei et al., 2013; Fraga et al., 2014; Yang et al., 2014; Lu et al., 2015; Polat et al., 2015; Shen et al., 2015; Xian et al., 2015; Ceylan et al., 2016; Diler and Oden, 2016). The study selection processes were presented in Figure 1.

**Table 1:** Detail characteristics of enrolled studies.

**SNP**	**References**	**Ethnicity**	**Source of control**	**Cancer type**	**Case**			**Control**			**Y(HWE)**
					**AA**	**AB**	**BB**	**AA**	**AB**	**BB**	
VEGF-rs10434	Abe et al., 2002	Asian	HB	RCC	113	31	1	109	33	3	Y
	Shen et al., 2015	Asian	HB	RCC	152	170	39	166	164	30	Y
	Lu et al., 2015	Asian	HB	RCC	172	191	49	365	375	85	Y
VEGF-rs1570360	McCarron et al., 2002	Caucasian	PB	PCa	114	109	15	120	109	34	Y
	Sfar et al., 2006	Caucasian	HB	PCa	58	37	6	36	50	14	Y
	Garcia-Closas et al., 2007	Caucasian	HB	BCa	431	383	78	389	407	82	Y
	Jacobs et al., 2008	Caucasian	PB	PCa	557	489	126	210	194	54	Y
	Ricketts et al., 2009	Caucasian	PB	RCC	134	143	47	146	130	38	Y
	Bruyère et al., 2010	Caucasian	PB	RCC	27	17	5	94	83	25	Y
	Yang et al., 2014	Asian	HB	BCa	224	187	69	213	162	45	Y
	Xian et al., 2015	Asian	HB	RCC	115	112	39	232	220	80	N
VEGF-rs2010963	Sfar et al., 2006	Caucasian	HB	PCa	29	57	15	44	46	10	Y
	Garcia-Closas et al., 2007	Caucasian	HB	BCa	388	395	98	387	396	93	Y
	Bruyère et al., 2010	Caucasian	PB	RCC	15	25	8	86	92	20	Y
	Sáenz-López et al., 2013	Caucasian	PB	RCC	101	93	20	129	118	32	Y
	Qin et al., 2014	Asian	HB	RCC	287	391	146	410	429	144	Y
	Shen et al., 2015	Asian	HB	RCC	121	170	69	134	163	63	Y
	Lu et al., 2015	Asian	HB	RCC	139	194	79	299	377	148	Y
	Xian et al., 2015	Asian	HB	RCC	30	132	104	49	256	227	Y
VEGF-rs3025039	Abe et al., 2002	Asian	HB	RCC	97	41	7	90	52	3	Y
	Sfar et al., 2006	Caucasian	HB	PCa	79	20	2	72	27	1	Y
	Garcia-Closas et al., 2007	Caucasian	HB	BCa	852	217	17	787	385	11	N
	Bruyère et al., 2010	Caucasian	PB	RCC	29	17	1	141	53	2	Y
	Sáenz-López et al., 2013	Caucasian	PB	RCC	156	57	2	200	73	7	Y
	Wang et al., 2013	Asian	HB	BCa	293	153	24	539	275	36	Y
	Yang et al., 2014	Asian	HB	BCa	307	149	24	284	121	15	Y
	Shen et al., 2015	Asian	HB	RCC	224	81	55	240	73	46	N
	Lu et al., 2015	Asian	HB	RCC	262	91	59	554	166	105	N
	Xian et al., 2015	Asian	HB	RCC	70	127	69	196	236	100	Y
VEGF-rs699947	Kim et al., 2005	Asian	HB	BCa	13	69	71	11	69	73	Y
	Garcia-Closas et al., 2007	Caucasian	HB	BCa	261	471	220	268	447	214	Y
	VanCleave et al., 2010	Mixed	PB	PCa	125	53	12	402	198	35	Y
	Ajaz et al., 2011	Asian	NA	RCC	30	81	32	44	41	21	Y
	Henríquez-Hernández et al., 2012	Caucasian	HB	BCa	11	25	23	14	16	13	Y
	Sáenz-López et al., 2013	Caucasian	PB	RCC	54	114	48	77	142	53	Y
	Ianni et al., 2013	Caucasian	HB	PCa	115	54	55	75	57	24	N
	Jaiswal et al., 2013	Asian	HB	BCa	67	116	17	106	112	32	Y
	Martinez-Fierro et al., 2013	Caucasian	HB	PCa	37	38	2	70	78	24	Y
	Shen et al., 2015	Asian	HB	RCC	150	149	61	178	141	41	Y
	Lu et al., 2015	Asian	HB	RCC	171	174	67	397	332	95	N
	Xian et al., 2015	Asian	HB	RCC	99	119	48	243	225	64	Y
VEGF-rs833061	Lin et al., 2003	Asian	HB	PCa	60	32	4	43	72	4	N
	Fukuda et al., 2007	Asian	HB	PCa	143	103	24	132	97	23	Y
	Garcia-Closas et al., 2007	Caucasian	HB	BCa	237	434	216	243	432	198	Y
	Onen et al., 2008	Mixed	PB	PCa	33	89	11	50	94	13	N
	Bruyère et al., 2010	Caucasian	PB	RCC	19	29	1	47	109	46	Y
	Sáenz-López et al., 2013	Caucasian	PB	RCC	56	111	49	77	138	58	Y
	Wang et al., 2013	Asian	HB	BCa	255	178	37	475	307	68	Y
	Lu et al., 2015	Asian	HB	RCC	228	93	91	513	168	143	N
HIF1α-rs11549465	Clifford et al., 2001	Caucasian	HB	RCC	42	6	0.1	110	27	6	N
	Chau et al., 2005	Mixed	NA	PCa	161	29	6	179	14	3	N
	Orr-Urtreger et al., 2007	Caucasian	PB	PCa	287	99	16	217	80	3	Y
	Li et al., 2007	Mixed	PB	PCa	818	209	14	995	221	18	Y
	Jacobs et al., 2008	Mixed	PB	PCa	1156	252	12	1138	284	28	N
	Nadaoka et al., 2008	Asian	HB	BCa	197	21	1	419	42	0.1	Y
	Foley et al., 2009	Caucasian	PB	PCa	65	30	0.1	175	13	0.1	Y
	Morris et al., 2009	Caucasian	PB	RCC	290	39	3	262	46	5	Y
	Li et al., 2012	Asian	HB	PCa	612	48	2	659	57	0.1	Y
	Qin et al., 2012	Asian	HB	RCC	572	46	2	578	43	2	Y
	Fraga et al., 2014	Caucasian	HB	PCa	579	164	11	566	156	14	Y
HIF1α-rs11549467	Clifford et al., 2001	Caucasian	HB	RCC	47	1	0.1	140	4	0.1	Y
	Chau et al., 2005	Mixed	NA	PCa	195	1	0.1	196	0.1	0.1	N
	Orr-Urtreger et al., 2007	Caucasian	PB	PCa	198	2	0.1	298	2	0.1	Y
	Li et al., 2007	Mixed	PB	PCa	1053	13	0.1	1247	17	0.1	Y
	Nadaoka et al., 2008	Asian	HB	BCa	204	13	2	421	40	0.1	Y
	Morris et al., 2009	Caucasian	PB	RCC	313	10	2	294	15	0.1	Y
	Li et al., 2012	Asian	HB	PCa	614	47	1	685	31	0.1	Y
	Qin et al., 2012	Asian	HB	RCC	575	45	0.1	584	39	0.1	Y
eNOS-rs1799983	Medeiros et al., 2002	Caucasian	HB	PCa	49	61	15	70	65	18	Y
	Marangoni et al., 2006	Caucasian	HB	PCa	30	50	4	30	29	6	Y
	Jacobs et al., 2008	Caucasian	PB	PCa	659	632	129	682	600	164	Y
	Lee et al., 2009	Caucasian	PB	PCa	517	468	103	607	557	129	Y
	Lee et al., 2009	Mixed	PB	PCa	77	20	0.1	280	88	5	Y
	Ryk et al., 2011	Caucasian	PB	BCa	128	106	28	75	62	13	Y
	Ziaei et al., 2013	Caucasian	HB	PCa	44	23	11	48	33	6	Y
	Safarinejad et al., 2013	Caucasian	HB	PCa	120	48	2	248	89	3	Y
	Verim et al., 2013	Caucasian	HB	BCa	7	49	10	31	44	13	Y
	Brankovic et al., 2013	Caucasian	HB	PCa	76	65	9	54	40	6	Y
	Polat et al., 2015	Caucasian	PB	BCa	7	59	9	48	75	20	Y
	Ceylan et al., 2016	Caucasian	HB	PCa	46	23	9	47	23	5	Y
	Diler et al., 2016	Caucasian	PB	PCa	6	55	23	65	41	10	Y
eNOS-rs2070744	Ryk et al., 2011	Caucasian	PB	BCa	152	142	40	84	63	8	Y
	Safarinejad et al., 2013	Caucasian	HB	PCa	52	93	25	150	159	31	Y
	Brankovic et al., 2013	Caucasian	HB	PCa	54	68	28	34	51	15	Y
	Polat et al., 2015	Caucasian	PB	BCa	24	40	11	56	72	15	Y
	Diler et al., 2016	Caucasian	PB	PCa	30	30	24	47	56	13	Y
eNOS-Intron 4a/b VNTR	Medeiros et al., 2002	Caucasian	HB	PCa	87	32	6	121	29	3	Y
	Sanli et al., 2011	Caucasian	HB	PCa	87	40	5	104	48	6	Y
	Amasyali et al., 2012	Caucasian	HB	BCa	52	63	8	137	59	5	Y
	Safarinejad et al., 2013	Caucasian	HB	PCa	101	54	15	249	88	3	Y
	Polat et al., 2015	Caucasian	PB	BCa	50	24	1	97	43	3	Y
	Diler et al., 2016	Caucasian	PB	PCa	65	16	3	83	31	2	Y
HRAS-rs12628	Johne et al., 2003	Caucasian	HB	BCa	151	119	42	164	170	26	N
	Sanyal et al., 2004	Caucasian	PB	BCa	153	147	2	54	61	6	N
	Traczyk et al., 2012	Caucasian	PB	BCa	45	64	23	49	48	9	Y
	Pandith et al., 2013	Asian	HB	BCa	90	42	8	135	25	0.1	Y

*SNP, single nucleotide polymorphism; HB, hospital-based; PB: population-based; RCC, renal cell carcinoma; PCa, prostate cancer; BCa, bladder cancer; HWE, Hardy Weinberg equilibrium; Y, controls conformed to HWE; N, controls were not conformed to HWE; Mixed, more than two ethnicities*.

For polymorphisms in VEGF gene (VEGF-rs10434, VEGF-rs1570360, VEGF-rs2010963, VEGF-rs3025039, VEGF-rs699947, VEGF-rs833061), a total of 49 case-control studies with 8,070 cases and 10,024 controls have met the inclusion criteria. Specifically, there are 918 cases and 1,330 controls in VEGF-rs10434, 3,522 cases and 3,167 controls in VEGF-rs1570360, 3,106 cases and 4,152 controls in VEGF-rs2010963, 3,582 cases and 4,890 controls in VEGF-rs3025039, 3,252 cases and 4,432 controls in VEGF-rs699947, 2,533 cases and 3,550 controls in VEGF-rs833061, respectively. Twenty-two studies of them were performed in Caucasians, 25 studies in Asians, and others were in mixed ethnic groups (including at least one race). Controls of 35 studies were hospital-based (H-B) and 13 studies were population-based (P-B), only one study didn't show whether it was H-B or P-B. Additionally, the distributions of polymorphisms in VEGF for control groups were consistent with HWE, except for these studies listed here (Lin et al., 2003; Garcia-Closas et al., 2007; Onen et al., 2008; Ianni et al., 2013; Lu et al., 2015; Shen et al., 2015; Xian et al., 2015).

For HIF1α-rs11549465 and rs11549467 polymorphisms, 19 eligible studies comprising 6,064 cases and 6,784 controls were enrolled. There are 5789 cases and 6,360 controls in HIF1α-rs11549465, 3,336 cases and 4,013 controls in HIF1α-rs11549467, respectively. Of them, eight studies were performed on subjects in Caucasians, six in Asians and the other five were in mixed ethnic groups. Moreover, nine studies were H-B, eight were P-B and the other two did not show H-B or P-B. There were several studies not consistent with HWE (Clifford et al., 2001; Chau et al., 2005; Jacobs et al., 2008).

For polymorphisms in eNOS gene (eNOS-rs1799983, eNOS-rs2070744, and eNOS-Intron 4a/b VNTR), 24 case-control studies with 4,286 cases and 5,009 controls were included in the current work. Specifically, there are 3,777 cases and 4,429 controls in eNOS-rs1799983, 813 cases, and 854 controls in eNOS-rs2070744, 709 cases and 1,111 controls in eNOS-Intron 4a/b VNTR, respectively. Of them, 23 studies were performed in Caucasian, 1 was in mixed ethnic groups. In addition, among these studies, 13 were H-B, and 11 were P-B. All the studies were consistent with HWE. While for HRAS-rs12628 polymorphism, finally, 4 eligible case-control studies comprising 886 cases and 747 controls were included. Of them, 3 studies were performed in Caucasian, 1 in Asian. Of these studies, 2 were H-B, and 2 were P-B. Two studies were not consistent with HWE (Johne et al., 2003; Sanyal et al., 2004).

### Quantitative synthesis

Table 2 and Supplementary Table 3 listed the main results of current meta-analysis work of polymorphisms in VEGF/hypoxia/angiogenesis pathway genes and risk of urogenital carcinomas.

**Table 2:** Representative results of meta-analysis for polymorphisms in VEGF/ Hypoxia/Angiogenesis genes and risk of Urogenital Carcinomas.

**SNP**	**Comparison**	**Subgroup**	**N**	***P*_H_**	***P*_A_**	**Random**	**Fixed**
eNOS-rs2070744	B vs. A	Overall	5	0.471	2.566E-05^**^	1.379 (1.188–1.602)	1.379 (1.187–1.602)
	B vs. A	PB	3	0.746	**6.260E-04**	1.436 (1.167–1.768)	1.437 (1.167–1.768)
	B vs. A	BCa	2	0.599	7.782E-03^*^	1.387 (1.089–1.767)	1.388 (1.090–1.768)
	B vs. A	PCa	3	0.196	1.113E-03^*^	1.362 (1.060–1.751)	1.373 (1.135–1.662)
	BA+BB vs. AA	BCa	2	0.922	3.913E-02^*^	1.402 (1.017–1.932)	1.402 (1.017–1.932)
	BA+BB vs. AA	PCa	3	0.125	2.278E-02^*^	1.307 (0.865–1.974)	1.375 (1.045–1.808)
	BB vs. AA	Overall	5	0.466	1.814E-05^**^	2.082 (1.481–2.926)	2.097 (1.495–2.942)
	BB vs. AA	PB	3	0.657	2.220E-04^**^	2.453 (1.510–3.985)	2.481 (1.532–4.019)
	BB vs. AA	BCa	2	0.438	6.251E-03^*^	2.241 (1.225–4.099)	2.298 (1.266–4.172)
	BB vs. AA	PCa	3	0.235	9.759E-04^*^	2.001 (1.211–3.306)	2.000 (1.325–3.018)
	BB vs. BA+AA	Overall	5	0.414	5.468E-05^**^	1.876 (1.370–2.567)	1.898 (1.390–2.591)
	BB vs. BA+AA	PB	3	0.393	1.915E-04^**^	2.331 (1.481–3.668)	2.352 (1.501–3.687)
	BB vs. BA+AA	BCa	2	0.358	1.503E-02^*^	1.946 (1.099–3.446)	2.006 (1.145–3.516)
	BB vs. BA+AA	PCa	3	0.214	1.300E-03^*^	1.869 (1.166–2.997)	1.848 (1.271–2.687)
eNOS-Intron 4a/b VNTR	BB vs. BA+AA	Overall	6	0.109	1.970E-04^**^	2.436 (1.109–5.351)	2.725 (1.608–4.619)
	BB vs. AA	BCa	2	0.150	4.899E-02^*^	2.157 (0.367–12.659)	2.661 (1.004–7.050)
eNOS-rs1799983	B vs. A	BCa	3	0.190	1.078E-02^*^	1.361 (1.024–1.809)	1.324 (1.067–1.642)
	BB vs. AA	BCa	3	0.229	2.056E-02^*^	2.070 (1.049–4.085)	1.887 (1.103–3.230)
HIF1α-rs11549467	B vs. A	PCa	4	0.490	4.413E-02^*^	1.461 (1.003–2.128)	1.465 (1.010–2.124)
HRAS-rs12628	BA+BB vs. AA	Y	2	0.127	3.647E-05^**^	2.220 (1.245–3.960)	2.211 (1.517–3.222)
	BB vs. AA	Y	2	0.163	5.725E-04^**^	7.207 (0.158–328.963)	4.174 (1.851–9.412)
	BB vs. BA+AA	HB	2	0.176	5.642E-04^**^	4.887 (0.138–172.923)	2.396 (1.458–3.938)
VEGF-rs2010963	BA vs. AA	RCC	6	0.530	1.762E-02^*^	1.168 (1.027–1.327)	1.168 (1.027–1.328)
	BA+BB vs. AA	RCC	6	0.240	6.790E-03^*^	1.158 (0.996–1.346)	1.181 (1.047–1.333)
	BB vs. AA	RCC	6	0.131	3.784E-02^*^	1.156 (0.910–1.468)	1.196 (1.010–1.416)
VEGF-rs3025039	B vs. A	Asian	6	0.337	4.545E-04^**^	1.179 (1.067–1.303)	1.180 (1.076–1.294)
	B vs. A	RCC	6	0.195	1.199E-03^*^	1.183 (1.028–1.363)	1.198 (1.074–1.336)
	BA+BB vs. AA	RCC	6	0.148	9.705E-03^*^	1.194 (0.986–1.446)	1.205 (1.046–1.389)
	BB vs. AA	Asian	6	0.548	7.532E-04^**^	1.400 (1.149–1.705)	1.401 (1.152–1.705)
	BB vs. AA	HB	8	0.773	4.316E-04^**^	1.404 (1.160–1.698)	1.405 (1.163–1.699)
	BB vs. AA	RCC	6	0.244	3.699E-03^*^	1.394 (1.043–1.863)	1.383 (1.111–1.721)
	BB vs. BA+AA	RCC	6	0.455	1.930E-02^*^	1.286 (1.046–1.582)	1.278 (1.041–1.570)
VEGF-rs699947	B vs. A	Asian	6	0.286	9.201E-07^**^	1.273 (1.139–1.424)	1.277 (1.158–1.408)
	B vs. A	RCC	5	0.603	1.311E-07^**^	1.311 (1.186–1.451)	1.312 (1.186–1.450)
	BA vs. AA	Asian	6	0.102	6.730E-05^**^	1.395 (1.122–1.734)	1.352 (1.166–1.568)
	BA vs. AA	RCC	5	0.109	5.669E-04^**^	1.347 (1.081–1.678)	1.306 (1.122–1.521)
	BA+BB vs. AA	Asian	6	0.221	1.339E-06^**^	1.427 (1.198–1.700)	1.410 (1.226–1.620)
	BA+BB vs. AA	RCC	5	0.189	4.602E-06^**^	1.417 (1.179–1.703)	1.394 (1.209–1.607)
	BB vs. AA	Asian	6	0.201	1.706E-05^**^	1.540 (1.185–2.003)	1.572 (1.279–1.931)
	BB vs. AA	RCC	5	0.772	7.220E-07^**^	1.688 (1.372–2.076)	1.687 (1.372–2.075)
	BB vs. BA+AA	RCC	5	0.780	1.791E-04^**^	1.436 (1.189–1.735)	1.435 (1.188–1.733)

SNP, single nucleotide polymorphism; P_H_, P value of Q test for heterogeneity test; P_A_, P < 0.05 was considered as statistically significant (bold font mark^*^) for cancer type subgroup analysis. And multiple testing P value according to Bonferroni correction [P < 0.05 / (12 polymorphisms × 5 models)] was considered as statistically significant (bold font mark^**^); PCa, prostate cancer; RCC, renal cell carcinoma; BCa, bladder cancer; HB, hospital based; PB, population based; HWE, Hardy Weinberg equilibrium. Heterogeneity was considered significant when the P-value < 0.1. A fixed effects model (Der-Simonian Laird) was used if there was no significant heterogeneity; otherwise, a random effects model (Der-Simonian Laird) was used.

### eNOS-rs2070744

The pooled results of five included studies had shown eNOS-rs2070744 polymorphism conferred a significantly higher overall risk to urogenital carcinomas in allele, homozygote and recessive models (B vs. A: *OR* = 1.379, 95%CI = 1.187–1.602, *P*_*A*_ = 2.566E-05; BB vs. AA: *OR* = 2.097, 95%CI = 1.495–2.942, *P*_*A*_ = 1.814E-05 and BB vs. BA + AA: *OR* = 1.898, 95%CI = 1.390–2.591, *P*_*A*_ = 5.468E-05), respectively. In the stratification analysis by source of control, an increased risk of urogenital neoplasms was also identified for P-B groups in allele, homozygote, and recessive models (B vs. A: *OR* = 1.437, 95%CI = 1.167–1.768, *P*_*A*_ = 6.260E-04; BB vs. AA: *OR* = 2.481, 95%CI = 1.532–4.019, *P*_*A*_ = 2.220E-04 and BB vs. BA + AA: *OR* = 2.352, 95%CI = 1.501–3.687, *P*_*A*_ = 1.915E-04). Moreover, when the stratification analysis conducted by cancer type (*P*_*A*_ value < 0.05, without Bonferroni correction), we also identified an increased risk of BCa in allelic, dominant, homozygote and recessive models (B vs. A: *OR* = 1.388, 95%CI = 1.090–1.768, *P*_*A*_ = 7.782E-03; BA+BB vs. AA: *OR* = 1.402, 95%CI = 1.017–1.932, *P*_*A*_ = 3.913E-02; BB vs. AA: *OR* = 2.298, 95%CI = 1.266–4.172, *P*_*A*_ = 6.251E-03 and BB vs. BA + AA: *OR* = 2.006, 95%CI = 1.145–3.516, *P*_*A*_ = 1.503E-02). An increased risk of PCa in allelic, dominant, homozygote, and recessive models (B vs. A: *OR* = 1.373, 95%CI = 1.135–1.662, *P*_*A*_ = 1.113E-03; BA+BB vs. AA: *OR* = 1.375, 95%CI = 1.045–1.808, *P*_*A*_ = 2.278E-02; BB vs. AA: *OR* = 2.000, 95%CI = 1.325–3.018, *P*_*A*_ = 9.759E-04 and BB vs. BA + AA: *OR* = 1.848, 95%CI = 1.271–2.687, *P*_*A*_ = 1.300E-03).

### eNOS-intron 4a/b VNTR

For eNOS-Intron 4a/b VNTR polymorphism, a total of six eligible case-control studies were included. The final analysis has shown that eNOS-Intron 4a/b VNTR polymorphism was related to an increased risk of urogenital neoplasms in recessive model (BB vs. BA + AA: *OR* = 2.725, 95%CI = 1.608–4.619, *P*_A_ = 1.970E-04). Subgroups analysis (*P*_*A*_ value without Bonferroni correction) identified an increased risk of BCa in homozygote models (BB vs. AA: *OR* = 2.661, 95%CI = 1.004–7.050, *P*_*A*_ = 4.899E-02).

### eNOS-rs1799983

Overall, there was no significant association between eNOS-rs1799983 polymorphism and the risk of urogenital neoplasms. However, subgroups analysis by cancer type revealed an increased risk of BCa in allelic and heterozygote models (B vs. A: *OR* = 1.324, 95%CI = 1.067–1.642, *P*_A_ = 1.078E-02; BB vs. AA: *OR* = 1.887, 95%CI = 1.103–3.230, *P*_A_ = 2.056E-02) (*P*_*A*_ value without Bonferroni correction).

### HIF1a-rs11549467

Overall, there was no significant association between HIF1a-rs11549467 polymorphism and the risk of urogenital neoplasms. Subgroups analysis by cancer type revealed an increased risk of PCa in allelic and heterozygote models (B vs. A: *OR* = 1.465, 95%CI = 1.010–2.124, *P*_A_ = 4.413E-02) (*P*_*A*_ value without Bonferroni correction).

### HRAS-rs12628

No significant association was uncovered for the association between HRAS-rs12628 polymorphism and urogenital carcinomas risk. However, when conducting the stratification analysis by HWE status, we identified an increased risk of urogenital carcinomas (all are BCa studies) in dominant and homozygote model for HWE (Y) groups (BB + BA vs. AA: *OR* = 2.211, 95%CI = 1.517–3.222, *P*_*A*_ = 3.647E-05; BB vs. AA: *OR* = 4.174, 95%CI = 1.851–9.412, *P*_*A*_ = 5.725E-04). Moreover, in the stratification analysis by source of control, an increased risk of urogenital carcinomas for H-B groups was also found (BB vs. BA+AA: *OR* = 2.396, 95%CI = 1.458–3.938, *P*_*A*_ = 5.642E-04).

### VEGF-rs2010963

Overall, there was no significant association between eNOS-rs1799983 polymorphism and the risk of urogenital neoplasms. Nevertheless, subgroups analysis by cancer type revealed an increased risk of RCC in heterozygote, dominant, and homozygote models (BA vs. AA: *OR* = 1.168, 95%CI = 1.027–1.328, *P*_*A*_ = 1.762E-02; BA+BB vs. AA: *OR* = 1.181, 95%CI = 1.047–1.333, *P*_*A*_ = 6.790E-03; BB vs. AA: *OR* = 1.196, 95%CI = 1.010–1.416, *P*_*A*_ = 3.784E-02) (*P*_*A*_ value without Bonferroni correction).

### VEGF-rs3025039

No significant association was found between VEGF-rs3025039 polymorphism and the risk of urogenital carcinomas. Nevertheless, subgroup analysis by ethnicity showed an increased risk of urogenital carcinomas in allelic and homozygote model for Asian population (B vs. A: *OR* = 1.180, 95%CI = 1.076–1.294, *P*_*A*_ = 4.545E-04; BB vs. AA: *OR* = 1.401, 95%CI = 1.152–1.705, *P*_*A*_ = 7.532E-04). Moreover, in the stratification analysis by source of control, an increased risk of urogenital carcinomas for H-B groups was also found (BB vs. AA: *OR* = 1.405, 95%CI = 1.163–1.699, *P*_*A*_ = 4.316E-04). Subgroups analysis by cancer type (*P*_*A*_ value without Bonferroni correction) revealed an increased risk of RCC in allelic, dominant, homozygote, and recessive models (B vs. A: *OR* = 1.198, 95%CI = 1.074–1.336, *P*_*A*_ = 1.199E-03; BA+BB vs. AA: *OR* = 1.205, 95%CI = 1.046–1.389, *P*_*A*_ = 9.705E-03; BB vs. AA: *OR* = 1.383, 95%CI = 1.111–1.721, *P*_*A*_ = 3.699E-03; BB vs. BA+AA: *OR* = 1.278, 95%CI = 1.041–1.570, *P*_*A*_ = 1.930E-02).

### VEGF-rs699947

Overall, there was no significant association between VEGF-rs699947 polymorphism and the risk of urogenital neoplasms. Nonetheless, an increased risk of urogenital carcinomas for Asian populations in allelic, heterozygote, dominant, and homozygote models were found in the stratification analysis by ethnicity (B vs. A: *OR* = 1.277, 95%CI = 1.158–1.408, *P*_*A*_ = 9.201E-07; BA vs. AA: *OR* = 1.352, 95%CI = 1.166–1.568, *P*_*A*_ = 6.730E-05; BB + BA vs. AA: *OR* = 1.410, 95%CI = 1.226–1.620, *P*_*A*_ = 1.339E-06 and BB vs. AA: *OR* = 1.572, 95%CI = 1.279–1.931, *P*_*A*_ = 1.706E-05). Moreover, when the stratification analysis conducted by cancer type, we also identified an increased risk of RCC in allelic, heterozygote, dominant, homozygote, and recessive models (B vs. A: *OR* = 1.312, 95%CI = 1.186–1.450, *P*_*A*_ = 1.311E-07; BA vs. AA: *OR* = 1.306, 95%CI = 1.122–1.521, *P*_*A*_ = 5.669E-04; BB + BA vs. AA: *OR* = 1.394, 95%CI = 1.209–1.607, *P*_*A*_ = 4.602E-06; BB vs. AA: *OR* = 1.687, 95%CI = 1.372–2.075, *P*_*A*_ = 7.220E-07 and BB vs. BA + AA: *OR* = 1.435, 95%CI = 1.188–1.733, *P*_*A*_ = 1.791E-04).

### VEGF-rs10434, VEGF-rs1570360, VEGF-rs833061, and HIF1a-rs11549465

There was no significant association between VEGF-rs10434, VEGF-rs1570360, VEGF-rs2010963, VEGF-rs833061, and HIF1α-rs11549465 polymorphisms and the risk of urogenital carcinomas. Furthermore, in the subgroup analysis by ethnicity, source of controls, cancer type or HWE status, similar results were also obtained.

### Sensitivity analysis and publication bias

In order to assess the stability of current meta-analysis result, sensitivity analysis was performed. The influence of the individual dataset on the pooled ORs was investigated after sequentially excluding each single case-control study. The study material alteration did not change the corresponding pooled ORs for all 12 polymorphisms (Supplementary Figure 1 and Supplementary Table 4). Furthermore, Begg's funnel plot and Egger's regression test were performed to evaluate the publication bias (Begg and Mazumdar, 1994; Egger et al., 1997). As for these 12 polymorphisms, no evidence of publication bias was identified by viewing the shape of Begg's funnel plot, which was further validated by Egger's regression test (Supplementary Figure 2 and Supplementary Table 5).

### LD analyses across populations

**Figure 2:**
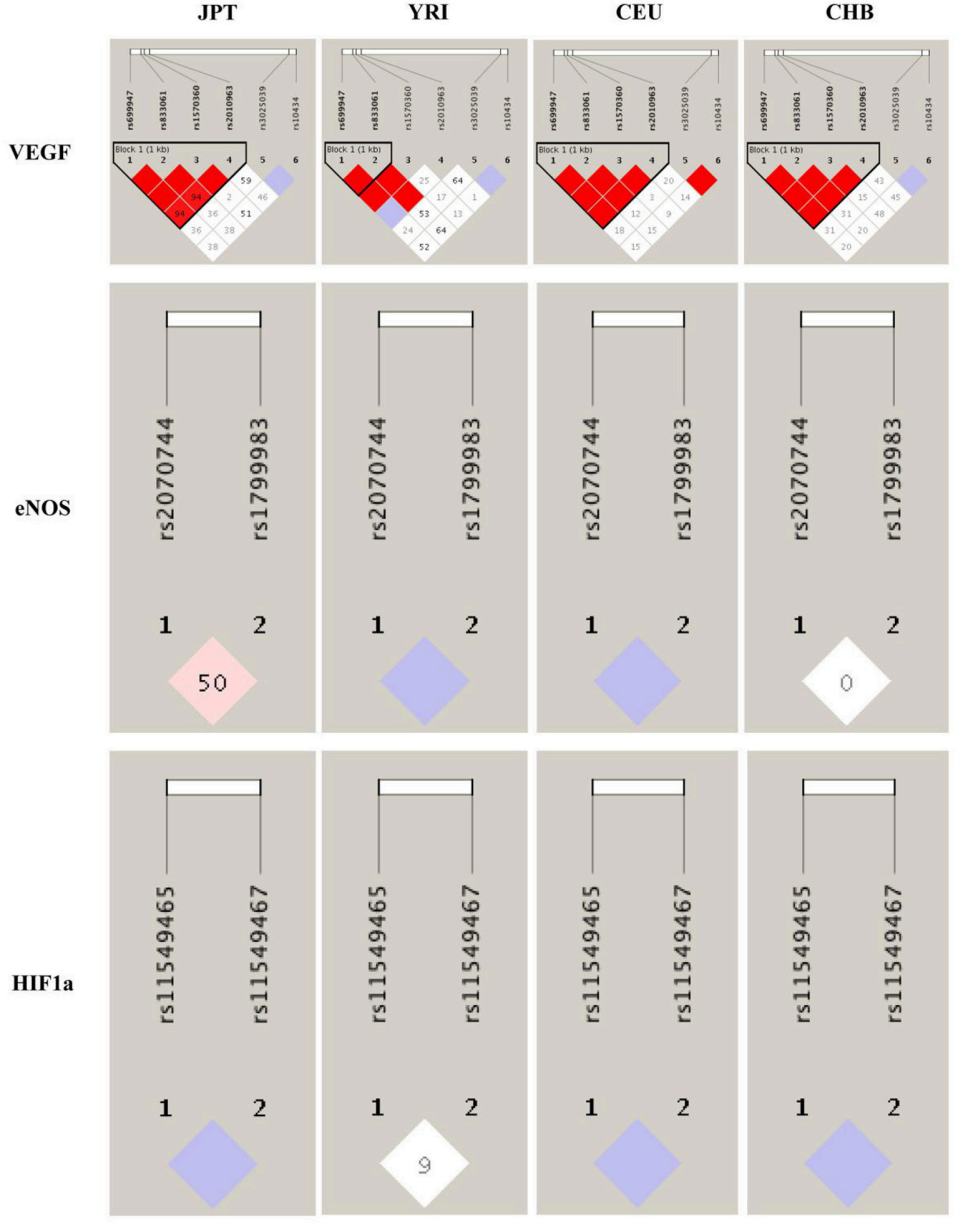
Linkage disequilibrium analyses for VEGF, eNOS, and HIF1α polymorphisms in populations from 1,000 genomes. The number of each cell represents *r*^2^ and white color cells shows no LD between polymorphisms. Population descriptors: JPT, Japanese in Tokyo, Japan; YRI, Yoruba in Ibadan, Nigeria; CEU, Utah residents with Northern and Western European ancestry from the CEPH collection; CHB, Han Chinese in Beijing, China.

To better understand the quantitative synthesis, we performed LD analysis to test for the existence of bins in the region comprising these polymorphisms of each angiogenesis related genes (VEGF-rs10434, VEGF-rs1570360, VEGF-rs2010963, VEGF-rs3025039, VEGF-rs699947, VEGF-rs833061, HIF1α-rs11549465, HIF1α-rs11549467, eNOS-rs1799983, and eNOS-rs2070744), respectively. LD plots for polymorphisms in each gene were presented in Figure 2 and Supplementary Table 6. Although we have uncovered significant LD for several polymorphisms in separate genes, such as VEGF, they were not statistically associated with urological neoplasms' risk in current work. As for the two significant polymorphisms (eNOS-Intron 4a/b VNTR and eNOS-rs2070744), LD analysis cannot be performed because eNOS-Intron 4a/b VNTR polymorphism was mismatched.

## Discussion

Angiogenesis is well-recognized as a key element for sustained tumor growth and a critical factor for tumor metastasis (McConkey et al., 2016; De Palma et al., 2017). Our previous studies also demonstrated upregulation of angiogenesis genes, e.g., VEGF played vital roles in bladder cancer progression (Zu et al., 2006; Lei et al., 2015; Zhou et al., 2015; Chen et al., 2016; Long et al., 2016). Other researchers also suggested that polymorphisms in angiogenesis pathway genes might be an important risk factors for the initiation and progression of urogenital neoplasms (Jacobs et al., 2008; Jaiswal et al., 2013; Orlandi et al., 2013; Dornbusch et al., 2016; Gong et al., 2017).

Some investigators have conducted case-control studies to evaluate the association between polymorphisms in angiogenesis related genes and the risk of urological tumors (Foley et al., 2009; Henríquez-Hernández et al., 2012; Li et al., 2012). However, most of prior studies addressed on limited polymorphisms in single angiogenesis related gene while neglected potential multiple gene's influence on urological carcinogenesis. Xian et al. found VEGF-rs3025039 genetic variant was associated with increased risk of RCC (Xian et al., 2015), but another study revealed no association (Abe et al., 2002). Li P et al. reported HIF1α-rs11549467 rather than HIF1α-rs11549465 polymorphism increased prostate cancer susceptibility (Li et al., 2012). Other studies failed to detect any association between these two polymorphisms and the risk of urinary cancers (Clifford et al., 2001; Morris et al., 2009). One study showed the eNOS-rs1799983 was associated with increased prostate cancer risk (Medeiros et al., 2002), while another study data suggested no association (Brankovic et al., 2013). HRAS-rs12628 polymorphism could mediate urinary bladder cancer development and predict tumors advancing (Amasyali et al., 2012), but another study showed no significant correlation (Sanyal et al., 2004). In the current study, we presented a comprehensive meta-analysis for 12 polymorphisms in four VEGF/hypoxia/angiogenesis pathway genes (VEGF, HIF1α, eNOS, and HRAS) including 96 case-control studies to examine the precise associations between these polymorphisms and risk of urogenital neoplasms.

Previous studies revealed that eNOS could modulate cancer-related events, such as VEGF-induced angiogenesis, invasion, and metastasis (Jadeski et al., 2000; Duda et al., 2004). The eNOS-rs2070744 polymorphism is a point mutation of thymine to cytosine (T>C) at −786 nucleotide in the 5′-flanking region of the eNOS gene (Nakayama et al., 1999). This mutation position in eNOS promoter region can alter gene activity and serum NO level, indicating the CC homozygote carriers may have an increased risk of cancer (Zhang et al., 2014). Present meta-analysis indicated that eNOS-rs2070744 polymorphism conferred a significantly increased overall risk to urogenital neoplasms. This is consistent with several previously published studies (Ryk et al., 2011; Brankovic et al., 2013; Safarinejad et al., 2013; Polat et al., 2015; Diler and Oden, 2016). The eNOS-Intron 4a/b VNTR polymorphism is a variable number of tandem repeats (27 nt) in intron 4 participating in basal plasma NO generation (Wang et al., 1997). Our final analysis results suggested that this polymorphism was related to an increased risk of urogenital neoplasms in recessive model. However, 6 case-control studies for eNOS-Intron 4a/b VNTR polymorphism enrolled in our study, there were two studies showed no significant relationship between this polymorphism and urogenital neoplasms (Sanli et al., 2011; Diler and Oden, 2016).

Although no significant association was uncovered between the association between VEGF-rs699947 polymorphism and the risk of urogenital neoplasms. In the subgroup analysis by ethnicity, we found an increased risk of urogenital neoplasms in Asian populations. Moreover, when the stratification analysis conducted by cancer type, we also identified an increased risk for RCC subtype. Similar results were found between VEGF-rs3025039 polymorphism and risk of urogenital neoplasms. Nevertheless, in the subgroup analysis of ethnicity, we observed an increased risk of urogenital neoplasms in allelic model for Asian populations. Moreover, in stratification analysis by source of control, an increased risk of urogenital neoplasms for H-B groups was also uncovered. Deviations in HWE status were influenced by methodological problems, such as the genotyping errors, the population stratification or selection bias, et al. Thus, we have conducted subgroup analyses by HWE status. For the HRAS-rs12628 polymorphism, we identified an increased risk of urogenital neoplasms in the dominant model for HWE (Y) groups.

While for VEGF (rs3025039, rs10434, rs1570360, rs2010963, and rs833061), HIF1α (rs11549465 and rs11549467), HRAS-rs12628 and eNOS-rs1799983 polymorphisms, our meta-analysis indicated there were no significant associations between them and urogenital neoplasms susceptibility even in subgroup analysis by ethnicity, HWE status, source of controls and cancer type (multiple testing *P*-value according to Bonferroni correction [*P* < 0.05/(12 polymorphisms × 5 models)] was considered as statistically significant). However, we also prefer to identify markers for specific tumors (eg. RCC, BCa, and PCa). For these SNPs, *P*_*A*_ < 0.05 was considered as statistically significant for cancer type subgroup analysis. Results suggested VEGF-rs699947, VEGF-rs3025039, and VEGF-rs2010963 polymorphisms may be a potential risk factor for RCC. And eNOS-rs2070744, eNOS-Intron 4a/b VNTR, eNOS-rs1799983, and HRAS-rs12628 polymorphisms may be a risk factor for BCa. In addition, eNOS-rs2070744 and HIF1a-rs11549467 polymorphisms may be a risk factor for PCa. Accordingly, these subgroups results suggested these SNPs might be used as potential diagnostic markers for RCC, BCa, and PCa, respectively. There are several important advantages for the current meta-analysis. First and foremost, we implemented a comprehensive database search to identify all eligible studies in the VEGF/hypoxia/angiogenesis pathway, making our meta-analysis more substantial and persuasive. Second, all included studies were assessed by Newcastle Ottawa Scale, aiming to exclude low quality studies and elevate the overall quality. Third, various subgroup analyses based on ethnicity, HWE status, source of controls and cancer type were performed, trying to further stratify the sources of heterogeneity. Fourth, in order to making our analysis more accurate, the recognized formula was performed to adjust the results. In addition, sensitivity analysis was applied to confirm the stability of included studies, and Begg's funnel plot and Egger's test was utilized to analyze publication bias.

However, several limitations should also be noted in our present meta-analysis. First, for several polymorphisms, particularly when the case number in the studies was small, it may result in an insufficient power for identifying weak association between these polymorphisms and urogenital neoplasms susceptibility. Second, we currently didn't validate these polymorphisms with related clinical consequences. In the follow-up study, we will focus on the functional aspects of how these polymorphisms affect genes expression and ultimately associate with tumorigenesis. Third, all of the included studies were restricted in English, exclusion of other languages studies may increase the publication bias. Fourth, we didn't consider several potential confounding factors in this study, such as the age, gender, smoking habit, drinking status, and environmental factors, etc. This limited us further study the genetic and environmental interaction model. Therefore, the current result should be cautious interpreted.

In summary, in conjunction with other studies, current meta-analysis results suggest that eNOS-rs2070744 and eNOS-Intron 4a/b VNTR polymorphisms are associated with elevated risk of urogenital carcinomas. In addition, VEGF-rs699947 polymorphism was also identified as an risk factor for renal carcinoma. Further well-designed case-control studies with large population size are warranted to strengthen our findings.

## Author contributions

J-BC, MZ, and X-BZ conceived and designed the study, performed the literature search and data extraction. J-BC, MZ, YC, P-HL, Y-WQ, CL, XC, W-BR, and Q-QL performed the meta-analysis and drafted the manuscript. L-FL, M-FC, H-QC, and X-BZ revised the manuscript. H-QC and X-BZ final approval of the version to be submitted.

### Conflict of interest statement

The authors declare that the research was conducted in the absence of any commercial or financial relationships that could be construed as a potential conflict of interest.

## Supplementary material

Supplementary Figure 1 Sensitivity analysis for VEGF-rs10434 **(A)**, VEGF-rs1570360 b, VEGF-rs2010963 **(C)**, VEGF-rs3025039 **(D)**, VEGF-rs699947 **(E)**, VEGF-rs833061 **(F)**, HIF1α-rs11549465 **(G)**, HIF1α-rs11549467 **(H)**, eNOS-rs1799983 **(I)**, eNOS-rs2070744 **(J)**, eNOS-Intron 4a/b VNTR **(K)**, HRAS-rs12628 **(L)** polymorphism and the risk of urogenital carcinomas (allelic comparison B vs. A).

Supplementary Figure 2 Begg's funnel plot for publication bias for VEGF-rs10434 **(A)**, VEGF-rs1570360 **(B)**, VEGF-rs2010963 **(C)**, VEGF-rs3025039 **(D)**, VEGF-rs699947 **(E)**, VEGF-rs833061 **(F)**, HIF1α-rs11549465 **(G)**, HIF1α-rs11549467 **(H)**, eNOS-rs1799983 **(I)**, eNOS-rs2070744 **(J)**, eNOS-Intron 4a/b VNTR **(K)**, HRAS-rs12628 **(L)** polymorphism (allelic comparison B vs. A).

Supplementary Table 1 Search strategies for each gene and the finally eligible articles included.

Supplementary Table 2 Methodological quality of the included studies according to the Newcastle-Ottawa Scale.

Supplementary Table 3 Results of meta-analysis for polymorphisms in VEGF/ Hypoxia/Angiogenesis genes and risk of Urogenital Carcinomas.

Supplementary Table 4 Details of the sensitivity analyses for the polymorphisms in VEGF/hypoxia/angiogenesis genes and the risk of urogenital carcinomas.

Supplementary Table 5 *P*-values of the Egger' test.

Supplementary Table 6 Summary of LD analyses.
