# Supplementary figures and images for: Association Between 12 Polymorphisms of VEGF/Hypoxia/Angiogenesis Pathway Genes and Risk of Urogenital Carcinomas: A Meta-Analysis Based on Case-Control Studies
Source: Front Physiol. 2018 Jun 11;9:715. doi: 10.3389/fphys.2018.00715 (PMC6004409; doi:10.3389/fphys.2018.00715)

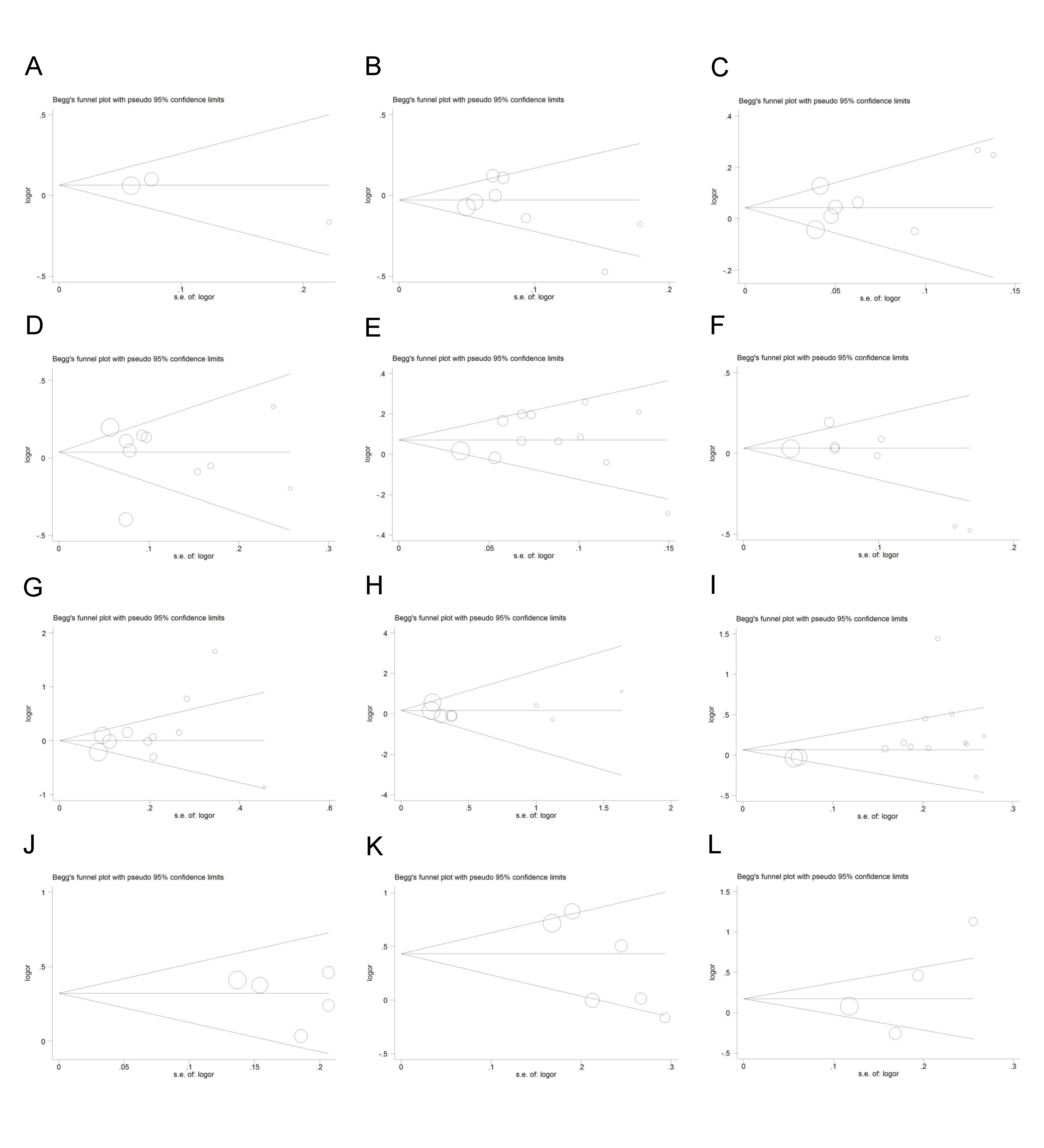

Supplement: Supplementary Figure 2 — Begg's funnel plot for publication bias for VEGF-rs10434 (A), VEGF-rs1570360 (B), VEGF-rs2010963 (C), VEGF-rs3025039 (D), VEGF-rs699947 (E), VEGF-rs833061 (F), HIF1α-rs11549465 (G), HIF1α-rs11549467 (H), eNOS-rs1799983 (I), eNOS-rs2070744 (J), eNOS-Intron 4a/b VNTR (K), HRAS-rs12628 (L) polymorphism (allelic comparison B vs. A). [file Image_2.TIFF]
