# Supplementary material for: Association Between 12 Polymorphisms of VEGF/Hypoxia/Angiogenesis Pathway Genes and Risk of Urogenital Carcinomas: A Meta-Analysis Based on Case-Control Studies
Source: Front Physiol. 2018 Jun 11;9:715. doi: 10.3389/fphys.2018.00715 (PMC6004409; doi:10.3389/fphys.2018.00715)
Supplement: Supplementary Table 1 — Search strategies for each gene and the finally eligible articles included. [file Table_1.DOC]

**Supplementary Table 1.** Search strategies for each gene and the finally eligible articles included.

| **Gene** | **Search strategy** | **Eligible Publications** | **Eligible Studies** |
| --- | --- | --- | --- |
| VEGF | (vascular endothelial growth factor OR VEGF) AND (polymorphism OR SNP OR mutation OR variant OR allele) AND (cancer OR carcinoma OR tumor OR neoplasm OR malignancy) | 24 | 49 |
| HIF1α | (hypoxia inducible factor-1α OR HIF-1A OR HIF1A OR HIF-1α OR HIF1α) AND (polymorphism OR SNP OR mutation OR variant OR allele) AND (cancer OR carcinoma OR tumor OR neoplasm OR malignancy) | 11 | 19 |
| eNOS | (endothelial nitric oxide synthase OR eNOS OR nitric oxide synthase 3 OR NOS3) AND (polymorphism OR SNP OR mutation OR variant OR allele) AND (cancer OR carcinoma OR tumor OR neoplasm OR malignancy) | 14 | 24 |
| HRAS | (HRas proto-oncogene OR HRAS OR H-RAS) AND (polymorphism OR SNP OR mutation OR variant OR allele) AND (cancer OR carcinoma OR tumor OR neoplasm OR malignancy) | 4 | 4 |

**Supplementary Table 2.** Methodological quality of the included studies according to the Newcastle-Ottawa Scale

| **Polymorphism** | **Author** | **Year** | **Adequacy of Case Definition** | **Representativeness of the Cases** | **Selection of Controls** | **Definition of Controls** | **Comparability Cases/Controls** | **Ascertainment of Exposure** | **Same Method of Ascertainment** | **Nonresponse rate** | **Total**  **scores** |
| --- | --- | --- | --- | --- | --- | --- | --- | --- | --- | --- | --- |
| VEGF-rs10434 | Abe et al. | 2002 | * | * | NA | * | ** | * | * | * | 8 |
|  | Shen et al. | 2015 | * | * | NA | * | ** | * | * | * | 8 |
|  | Lu et al. | 2015 | * | * | NA | * | ** | * | * | * | 8 |
| VEGF-rs1570360 | McCarron et al. | 2002 | * | * | NA | * | ** | * | * | * | 8 |
|  | Sfar et al. | 2006 | * | * | * | * | ** | * | * | * | 9 |
|  | Garcia-Closas et al. | 2007 | * | * | * | * | ** | * | * | * | 9 |
|  | Jacobs et al. | 2008 | * | * | * | * | ** | * | * | * | 9 |
|  | Ricketts et al. | 2009 | * | * | * | * | ** | * | * | * | 9 |
|  | Bruyère et al. | 2010 | * | * | * | * | ** | * | * | * | 9 |
|  | Yang et al. | 2014 | * | * | NA | * | ** | * | * | * | 8 |
|  | Xian et al. | 2015 | * | * | NA | * | ** | * | * | * | 8 |
| VEGF-rs2010963 | Sfar et al. | 2006 | * | * | * | * | ** | * | * | * | 9 |
|  | Garcia-Closas et al. | 2007 | * | * | * | * | ** | * | * | * | 9 |
|  | Bruyère et al. | 2010 | * | * | * | * | ** | * | * | * | 9 |
|  | Sáenz-López et al. | 2013 | * | * | * | * | ** | * | * | * | 9 |
|  | Qin et al. | 2014 | * | * | * | * | ** | * | * | * | 9 |
|  | Shen et al. | 2015 | * | * | NA | * | ** | * | * | * | 8 |
|  | Lu et al. | 2015 | * | * | NA | * | ** | * | * | * | 8 |
|  | Xian et al. | 2015 | * | * | NA | * | ** | * | * | * | 8 |
| VEGF-rs3025039 | Abe et al. | 2002 | * | * | NA | * | ** | * | * | * | 8 |
|  | Sfar et al. | 2006 | * | * | * | * | ** | * | * | * | 9 |
|  | Garcia-Closas et al. | 2007 | * | * | * | * | ** | * | * | * | 9 |
|  | Bruyère et al. | 2010 | * | * | * | * | ** | * | * | * | 9 |
|  | Sáenz-López et al. | 2013 | * | * | * | * | ** | * | * | * | 9 |
|  | Wang et al. | 2013 | * | * | NA | * | ** | * | * | * | 8 |
|  | Yang et al. | 2014 | * | * | NA | * | ** | * | * | * | 8 |
|  | Shen et al. | 2015 | * | * | NA | * | ** | * | * | * | 8 |
|  | Lu et al. | 2015 | * | * | NA | * | ** | * | * | * | 8 |
|  | Xian et al. | 2015 | * | * | NA | * | ** | * | * | * | 8 |
| VEGF-rs699947 | Kim et al. | 2005 | * | * | NA | * | ** | * | * | * | 8 |
|  | Garcia-Closas et al. | 2007 | * | * | * | * | ** | * | * | * | 9 |
|  | VanCleave et al. | 2010 | * | * | NA | * | ** | * | * | * | 8 |
|  | Ajaz et al. | 2011 | * | * | NA | NA | ** | * | * | * | 7 |
|  | Henríquez-Hernández et al. | 2012 | * | * | * | * | ** | * | * | * | 9 |
|  | Sáenz-López et al. | 2013 | * | * | * | * | ** | * | * | * | 9 |
|  | Ianni et al. | 2013 | * | * | NA | * | ** | * | * | * | 8 |
|  | Jaiswal et al. | 2013 | * | * | * | * | ** | * | * | * | 9 |
|  | Martinez-Fierro et al | 2013 | * | * | NA | * | ** | * | * | * | 8 |
|  | Shen et al. | 2015 | * | * | NA | * | ** | * | * | * | 8 |
|  | Lu et al. | 2015 | * | * | NA | * | ** | * | * | * | 8 |
|  | Xian et al. | 2015 | * | * | NA | * | ** | * | * | * | 8 |
| VEGF-rs833061 | Lin et al. | 2003 | * | * | NA | * | ** | * | * | * | 8 |
|  | Fukuda et al. | 2007 | * | * | NA | * | ** | * | * | * | 8 |
|  | Garcia-Closas et al. | 2007 | * | * | * | * | ** | * | * | * | 9 |
|  | Onen et al. | 2008 | * | * | * | * | ** | * | * | * | 9 |
|  | Bruyère et al. | 2010 | * | * | * | * | ** | * | * | * | 9 |
|  | Sáenz-López et al. | 2013 | * | * | * | * | ** | * | * | * | 9 |
|  | Wang et al. | 2013 | * | * | NA | * | ** | * | * | * | 8 |
|  | Lu et al. | 2015 | * | * | NA | * | ** | * | * | * | 8 |
| HIF1a-rs11549465 | Clifford et al. | 2001 | * | * | NA | * | ** | * | * | * | 8 |
|  | Chau et al. | 2005 | * | * | NA | NA | ** | * | * | * | 7 |
|  | Orr-Urtreger et al. | 2007 | * | * | NA | * | ** | * | * | * | 8 |
|  | Li et al. | 2007 | * | * | * | * | ** | * | * | * | 9 |
|  | Jacobs et al. | 2008 | * | * | * | * | ** | * | * | * | 9 |
|  | Nadaoka et al. | 2008 | * | * | NA | * | ** | * | * | * | 8 |
|  | Foley et al. | 2009 | * | * | NA | * | ** | * | * | * | 8 |
|  | Morris et al. | 2009 | * | * | NA | * | ** | * | * | * | 8 |
|  | Li et al. | 2012 | * | * | * | * | ** | * | * | * | 9 |
|  | Qin et al. | 2012 | * | * | * | * | ** | * | * | * | 9 |
|  | Fraga et al. | 2014 | * | * | NA | * | ** | * | * | * | 8 |
| HIF1a-rs11549467 | Clifford et al. | 2001 | * | * | NA | * | ** | * | * | * | 8 |
|  | Chau et al. | 2005 | * | * | NA | NA | ** | * | * | * | 7 |
|  | Orr-Urtreger et al. | 2007 | * | * | NA | * | ** | * | * | * | 8 |
|  | Li et al. | 2007 | * | * | * | * | ** | * | * | * | 9 |
|  | Nadaoka et al. | 2008 | * | * | NA | * | ** | * | * | * | 8 |
|  | Morris et al. | 2009 | * | * | NA | * | ** | * | * | * | 8 |
|  | Li et al. | 2012 | * | * | * | * | ** | * | * | * | 9 |
|  | Qin et al. | 2012 | * | * | * | * | ** | * | * | * | 9 |
| eNOS-rs1799983 | Medeiros et al. | 2002 | * | * | NA | * | ** | * | * | * | 8 |
|  | Marangoni et al. | 2006 | * | * | NA | * | ** | * | * | * | 8 |
|  | Jacobs et al. | 2008 | * | * | * | * | ** | * | * | * | 9 |
|  | Lee et al. | 2009 | * | * | * | * | ** | * | * | * | 9 |
|  | Ryk et al. | 2011 | * | * | NA | * | ** | * | * | * | 8 |
|  | Ziaei et al. | 2013 | * | * | NA | * | ** | * | * | * | 8 |
|  | Safarinejad et al. | 2013 | * | * | * | * | ** | * | * | * | 9 |
|  | Verim et al. | 2013 | * | * | NA | * | ** | * | * | * | 8 |
|  | Brankovic et al. | 2013 | * | * | * | * | ** | * | * | * | 9 |
|  | Polat et al. | 2015 | * | * | * | * | ** | * | * | * | 9 |
|  | Ceylan et al. | 2016 | * | * | NA | * | ** | * | * | * | 8 |
|  | Diler et al. | 2016 | * | * | NA | * | ** | * | * | * | 8 |
| eNOS-rs2070744 | Ryk et al. | 2011 | * | * | NA | * | ** | * | * | * | 8 |
|  | Safarinejad et al. | 2013 | * | * | * | * | ** | * | * | * | 9 |
|  | Brankovic et al. | 2013 | * | * | * | * | ** | * | * | * | 9 |
|  | Polat et al. | 2015 | * | * | * | * | ** | * | * | * | 9 |
|  | Diler et al. | 2016 | * | * | NA | * | ** | * | * | * | 8 |
| eNOS-Intron 4a/b VNTR | Medeiros et al. | 2002 | * | * | NA | * | ** | * | * | * | 8 |
|  | Sanli et al. | 2011 | * | * | * | * | ** | * | * | * | 9 |
|  | Amasyali et al. | 2012 | * | * | * | * | ** | * | * | * | 9 |
|  | Safarinejad et al. | 2013 | * | * | * | * | ** | * | * | * | 9 |
|  | Polat et al. | 2015 | * | * | * | * | ** | * | * | * | 9 |
|  | Diler et al. | 2016 | * | * | NA | * | ** | * | * | * | 8 |
| HRAS-rs12628 | Johne et al. | 2003 | * | * | NA | * | ** | * | * | * | 8 |
|  | Sanyal et al. | 2004 | * | * | NA | * | ** | * | * | * | 8 |
|  | Traczyk et al. | 2012 | * | * | NA | * | ** | * | * | * | 8 |
|  | Pandith et al. | 2013 | * | * | * | * | ** | * | * | * | 9 |

High quality choices with a ‘star’. A study can be awarded a maximum of one star for each numbered item within the Selection and Exposure categories. A maximum of two stars can be given for Comparability. NA: not applicable.

**Supplementary table 3.** Results of meta-analysis for polymorphisms in VEGF/ Hypoxia/Angiogenesis genes and risk of Urogenital Carcinomas.

| **SNP** | **Comparison** | **Subgroup** | **N** | ***P*_H_** | ***P*_A_** | **Random** | **Fixed** |
| --- | --- | --- | --- | --- | --- | --- | --- |
| eNOS-Intron 4a/b VNTR | B VS. A | Overall | 6 | 0.003 | 4.354E-02 | 1.416 (1.010-1.986) | 1.525 (1.284-1.811) |
|  | B VS. A | HB | 4 | 0.020 | 4.202E-03 | 1.687 (1.179-2.413) | 1.731 (1.427-2.100) |
|  | B VS. A | PB | 2 | 0.652 | 7.324E-01 | 0.935 (0.635-1.377) | 0.935 (0.635-1.376) |
|  | B VS. A | BCa | 2 | 0.013 | 2.761E-01 | 1.555 (0.703-3.440) | 1.726 (1.280-2.327) |
|  | B VS. A | PCa | 4 | 0.012 | 1.732E-01 | 1.338 (0.880-2.036) | 1.436 (1.164-1.771) |
|  | BA VS. AA | Overall | 6 | 0.008 | 1.385E-01 | 1.335 (0.911-1.956) | 1.405 (1.139-1.733) |
|  | BA VS. AA | HB | 4 | 0.032 | 2.647E-02 | 1.605 (1.057-2.437) | 1.614 (1.272-2.049) |
|  | BA VS. AA | PB | 2 | 0.287 | 5.388E-01 | 0.868 (0.535-1.408) | 0.868 (0.553-1.363) |
|  | BA VS. AA | BCa | 2 | 0.015 | 2.275E-01 | 1.778 (0.698-4.530) | 1.940 (1.342-2.804) |
|  | BA VS. AA | PCa | 4 | 0.146 | 1.585E-01 | 1.168 (0.818-1.667) | 1.203 (0.931-1.555) |
|  | BA+BB VS.AA | Overall | 6 | 0.003 | 7.799E-02 | 1.425 (0.961-2.114) | 1.523 (1.245-1.863) |
|  | BA+BB VS.AA | HB | 4 | 0.019 | 1.047E-02 | 1.744 (1.139-2.670) | 1.768 (1.407-2.223) |
|  | BA+BB VS.AA | PB | 2 | 0.423 | 6.145E-01 | 0.895 (0.577-1.388) | 0.893 (0.576-1.385) |
|  | BA+BB VS.AA | BCa | 2 | 0.008 | 2.548E-01 | 1.787 (0.658-4.850) | 1.976 (1.380-2.830) |
|  | BA+BB VS.AA | PCa | 4 | 0.045 | 2.521E-01 | 1.275 (0.841-1.933) | 1.351 (1.058-1.724) |
|  | BB VS. AA | Overall | 6 | 0.068 | 2.095E-02 | 2.720 (1.163-6.359) | 3.070 (1.805-5.223) |
|  | BB VS. AA | HB | 4 | 0.043 | 1.883E-02 | 3.465 (1.228-9.771) | 3.645 (2.022-6.569) |
|  | BB VS. AA | PB | 2 | 0.466 | 7.641E-01 | 1.258 (0.303-5.225) | 1.232 (0.316-4.799) |
|  | BB VS. AA | BCa | 2 | 0.150 | **4.899E-02^*^** | 2.157 (0.367-12.659) | 2.661 (1.004-7.050) |
|  | BB VS. AA | PCa | 4 | 0.041 | 7.273E-02 | 2.907 (0.906-9.325) | 3.241 (1.717-6.119) |
|  | BB VS.BA+AA | Overall | 6 | 0.109 | **1.970E-04^**^** | 2.436 (1.109-5.351) | 2.725 (1.608-4.619) |
|  | BB VS.BA+AA | HB | 4 | 0.061 | 3.159E-02 | 2.922 (1.099-7.767) | 3.123 (1.744-5.594) |
|  | BB VS.BA+AA | PB | 2 | 0.416 | 7.140E-01 | 1.323 (0.320-5.465) | 1.287 (0.334-4.949) |
|  | BB VS.BA+AA | BCa | 2 | 0.260 | 1.746E-01 | 1.852 (0.521-6.580) | 1.963 (0.741-5.196) |
|  | BB VS.BA+AA | PCa | 4 | 0.058 | 6.719E-02 | 2.799 (0.93-8.4240) | 3.103 (1.645-5.851) |
| eNOS-rs1799983 | B VS. A | Overall | 13 | 0.000 | 2.112E-02 | 1.235 (1.032-1.477) | 1.066 (0.996-1.141) |
|  | B VS. A | Caucasian | 12 | 0.000 | 1.058E-02 | 1.274 (1.058-1.534) | 1.073 (1.002-1.150) |
|  | B VS. A | HB | 7 | 0.874 | 2.647E-02 | 1.199 (1.021-1.409) | 1.200 (1.021-1.409) |
|  | B VS. A | PB | 6 | 0.000 | 1.053E-01 | 1.275 (0.950-1.712) | 1.039 (0.963-1.120) |
|  | B VS. A | BCa | 3 | 0.190 | **1.078E-02^*^** | 1.361 (1.024-1.809) | 1.324 (1.067-1.642) |
|  | B VS. A | PCa | 10 | 0.000 | 9.347E-02 | 1.196 (0.970-1.473) | 1.040 (0.968-1.118) |
|  | BA VS. AA | Overall | 13 | 0.000 | 8.729E-03 | 1.425 (1.094-1.856) | 1.155 (1.051-1.269) |
|  | BA VS. AA | Caucasian | 12 | 0.000 | 4.642E-03 | 1.499 (1.133-1.983) | 1.167 (1.061-1.285) |
|  | BA VS. AA | HB | 7 | 0.054 | 1.022E-01 | 1.313 (0.947-1.820) | 1.280 (1.032-1.587) |
|  | BA VS. AA | PB | 6 | 0.000 | 2.939E-02 | 1.584 (1.047-2.396) | 1.127 (1.015-1.252) |
|  | BA VS. AA | BCa | 3 | 0.000 | 1.036E-01 | 2.846 (0.808-10.023) | 1.810 (1.298-2.525) |
|  | BA VS. AA | PCa | 10 | 0.000 | 1.052E-01 | 1.233 (0.957-1.588) | 1.109 (1.004-1.223) |
|  | BA+BB VS.AA | Overall | 13 | 0.000 | 6.968E-03 | 1.428 (1.102-1.849) | 1.132 (1.034-1.238) |
|  | BA+BB VS.AA | Caucasian | 12 | 0.000 | 3.239E-03 | 1.507 (1.147-1.980) | 1.144 (1.044-1.253) |
|  | BA+BB VS.AA | HB | 7 | 0.140 | 1.442E-02 | 1.313 (0.998-1.728) | 1.295 (1.053-1.592) |
|  | BA+BB VS.AA | PB | 6 | 0.000 | 3.122E-02 | 1.577 (1.042-2.386) | 1.097 (0.992-1.212) |
|  | BA+BB VS.AA | BCa | 3 | 0.000 | 9.241E-02 | 2.722 (0.848-8.736) | 1.770 (1.286-2.438) |
|  | BA+BB VS.AA | PCa | 10 | 0.000 | 8.771E-02 | 1.248 (0.968-1.609) | 1.087 (0.990-1.194) |
|  | BB VS. AA | Overall | 13 | 0.000 | 2.929E-02 | 1.615 (1.049-2.485) | 1.079 (0.919-1.266) |
|  | BB VS. AA | Caucasian | 12 | 0.000 | 2.540E-02 | 1.639 (1.063-2.529) | 1.086 (0.925-1.275) |
|  | BB VS. AA | HB | 7 | 0.626 | 7.013E-02 | 1.466 (0.964-2.230) | 1.466 (0.969-2.217) |
|  | BB VS. AA | PB | 6 | 0.000 | 8.203E-02 | 1.834 (0.926-3.632) | 1.022 (0.858-1.216) |
|  | BB VS. AA | BCa | 3 | 0.229 | **2.056E-02^*^** | 2.070 (1.049-4.085) | 1.887 (1.103-3.230) |
|  | BB VS. AA | PCa | 10 | 0.000 | 1.324E-01 | 1.468 (0.890-2.419) | 1.019 (0.861-1.205) |
|  | BB VS.BA+AA | Overall | 13 | 0.051 | 4.603E-01 | 1.106 (0.846-1.447) | 0.963 (0.827-1.122) |
|  | BB VS.BA+AA | Caucasian | 12 | 0.041 | 4.344E-01 | 1.114 (0.849-1.462) | 0.969 (0.832-1.128) |
|  | BB VS.BA+AA | HB | 7 | 0.675 | 4.531E-01 | 1.154 (0.782-1.704) | 1.159 (0.789-1.702) |
|  | BB VS.BA+AA | PB | 6 | 0.007 | 5.906E-01 | 1.116 (0.749-1.662) | 0.931 (0.788-1.099) |
|  | BB VS.BA+AA | BCa | 3 | 0.762 | 8.006E-01 | 1.059 (0.670-1.676) | 1.060 (0.674-1.668) |
|  | BB VS.BA+AA | PCa | 10 | 0.016 | 4.043E-01 | 1.159 (0.819-1.640) | 0.951 (0.809-1.119) |
| eNOS-rs2070744 | B VS. A | Overall | 5 | 0.471 | **2.566E-05^**^** | 1.379 (1.188-1.602) | 1.379 (1.187-1.602) |
|  | B VS. A | HB | 2 | 0.103 | 1.223E-02 | 1.276 (0.884-1.840) | 1.318 (1.062-1.637) |
|  | B VS. A | PB | 3 | 0.746 | **6.260E-04** | 1.436 (1.167-1.768) | 1.437 (1.167-1.768) |
|  | B VS. A | BCa | 2 | 0.599 | **7.782E-03^*^** | 1.387 (1.089-1.767) | 1.388 (1.090-1.768) |
|  | B VS. A | PCa | 3 | 0.196 | **1.113E-03^*^** | 1.362 (1.060-1.751) | 1.373 (1.135-1.662) |
|  | BA VS. AA | Overall | 5 | 0.239 | 6.318E-02 | 1.203 (0.923-1.567) | 1.231 (0.989-1.532) |
|  | BA VS. AA | HB | 2 | 0.049 | 5.610E-01 | 1.224 (0.619-2.421) | 1.329 (0.960-1.841) |
|  | BA VS. AA | PB | 3 | 0.539 | 3.403E-01 | 1.154 (0.858-1.553) | 1.155 (0.859-1.552) |
|  | BA VS. AA | BCa | 2 | 0.915 | 1.751E-01 | 1.260 (0.902-1.761) | 1.260 (0.902-1.761) |
|  | BA VS. AA | PCa | 3 | 0.065 | 6.985E-01 | 1.105 (0.667-1.831) | 1.209 (0.905-1.614) |
|  | BA+BB VS.AA | Overall | 5 | 0.382 | 2.133E-03 | 1.380 (1.113-1.712) | 1.386 (1.125-1.707) |
|  | BA+BB VS.AA | HB | 2 | 0.046 | 4.148E-01 | 1.314 (0.682-2.531) | 1.420 (1.041-1.938) |
|  | BA+BB VS.AA | PB | 3 | 0.920 | 3.247E-02 | 1.359 (1.026-1.799) | 1.359 (1.026-1.799) |
|  | BA+BB VS.AA | BCa | 2 | 0.922 | **3.913E-02^*^** | 1.402 (1.017-1.932) | 1.402 (1.017-1.932) |
|  | BA+BB VS.AA | PCa | 3 | 0.125 | **2.278E-02^*^** | 1.307 (0.865-1.974) | 1.375 (1.045-1.808) |
|  | BB VS. AA | Overall | 5 | 0.466 | **1.814E-05^**^** | 2.082 (1.481-2.926) | 2.097 (1.495-2.942) |
|  | BB VS. AA | HB | 2 | 0.170 | 2.175E-02 | 1.718 (0.883-3.342) | 1.754 (1.085-2.834) |
|  | BB VS. AA | PB | 3 | 0.657 | **2.220E-04^**^** | 2.453 (1.510-3.985) | 2.481 (1.532-4.019) |
|  | BB VS. AA | BCa | 2 | 0.438 | **6.251E-03^*^** | 2.241 (1.225-4.099) | 2.298 (1.266-4.172) |
|  | BB VS. AA | PCa | 3 | 0.235 | **9.759E-04^*^** | 2.001 (1.211-3.306) | 2.000 (1.325-3.018) |
|  | BB VS.BA+AA | Overall | 5 | 0.414 | **5.468E-05^**^** | 1.876 (1.370-2.567) | 1.898 (1.390-2.591) |
|  | BB VS.BA+AA | HB | 2 | 0.538 | 5.702E-02 | 1.536 (0.994-2.373) | 1.529 (0.987-2.367) |
|  | BB VS.BA+AA | PB | 3 | 0.393 | **1.915E-04^**^** | 2.331 (1.481-3.668) | 2.352 (1.501-3.687) |
|  | BB VS.BA+AA | BCa | 2 | 0.358 | **1.503E-02^*^** | 1.946 (1.099-3.446) | 2.006 (1.145-3.516) |
|  | BB VS.BA+AA | PCa | 3 | 0.214 | **1.300E-03^*^** | 1.869 (1.166-2.997) | 1.848 (1.271-2.687) |
| HIF1α-rs11549465 | B VS. A | Overall | 11 | 0.000 | 3.349E-01 | 1.111 (0.897-1.376) | 1.010 (0.924-1.103) |
|  | B VS. A | Asian | 3 | 0.880 | 6.762E-01 | 1.054 (0.824-1.348) | 1.054 (0.824-1.348) |
|  | B VS. A | Caucasian | 5 | 0.000 | 5.979E-01 | 1.140 (0.701-1.854) | 1.054 (0.906-1.227) |
|  | B VS. A | Mixed | 3 | 0.001 | 5.050E-01 | 1.138 (0.778-1.665) | 0.972 (0.861-1.098) |
|  | B VS. A | HB | 5 | 0.404 | 7.929E-01 | 0.986 (0.840-1.159) | 0.979 (0.834-1.148) |
|  | B VS. A | PB | 5 | 0.000 | 3.598E-01 | 1.182 (0.827-1.689) | 0.992 (0.890-1.106) |
|  | B VS. A | N | 3 | 0.001 | 8.993E-01 | 0.952 (0.444-2.041) | 0.861 (0.737-1.007) |
|  | B VS. A | Y | 8 | 0.001 | 2.320E-01 | 1.149 (0.915-1.442) | 1.089 (0.978-1.212) |
|  | B VS. A | PCa | 7 | 0.000 | 1.042E-01 | 1.251 (0.955-1.638) | 1.032 (0.938-1.135) |
|  | B VS. A | RCC | 3 | 0.133 | 1.443E-01 | 0.786 (0.515-1.199) | 0.818 (0.624-1.071) |
|  | BA VS. AA | Overall | 11 | 0.000 | 3.246E-01 | 1.117 (0.896-1.392) | 1.033 (0.936-1.141) |
|  | BA VS. AA | Asian | 3 | 0.817 | 9.981E-01 | 1.001 (0.772-1.296) | 1.000 (0.772-1.296) |
|  | BA VS. AA | Caucasian | 5 | 0.000 | 5.117E-01 | 1.195 (0.702-2.034) | 1.061 (0.893-1.261) |
|  | BA VS. AA | Mixed | 3 | 0.009 | 4.178E-01 | 1.158 (0.812-1.651) | 1.025 (0.894-1.176) |
|  | BA VS. AA | HB | 5 | 0.793 | 9.402E-01 | 0.996 (0.835-1.187) | 0.993 (0.833-1.184) |
|  | BA VS. AA | PB | 5 | 0.000 | 3.473E-01 | 1.203 (0.818-1.768) | 1.023 (0.906-1.156) |
|  | BA VS. AA | N | 3 | 0.016 | 8.339E-01 | 1.076 (0.541-2.142) | 0.925 (0.775-1.104) |
|  | BA VS. AA | Y | 8 | 0.000 | 3.231E-01 | 1.140 (0.879-1.478) | 1.087 (0.964-1.225) |
|  | BA VS. AA | PCa | 7 | 0.000 | 1.308E-01 | 1.250 (0.936-1.669) | 1.055 (0.948-1.174) |
|  | BA VS. AA | RCC | 3 | 0.377 | 3.778E-01 | 0.879 (0.652-1.185) | 0.875 (0.651-1.177) |
|  | BA+BB VS.AA | Overall | 11 | 0.000 | 3.225E-01 | 1.120 (0.895-1.403) | 1.022 (0.928-1.126) |
|  | BA+BB VS.AA | Asian | 3 | 0.857 | 8.352E-01 | 1.028 (0.796-1.326) | 1.027 (0.796-1.326) |
|  | BA+BB VS.AA | Caucasian | 5 | 0.000 | 5.505E-01 | 1.176 (0.691-1.999) | 1.060 (0.896-1.253) |
|  | BA+BB VS.AA | Mixed | 3 | 0.002 | 4.526E-01 | 1.159 (0.788-1.704) | 0.998 (0.874-1.140) |
|  | BA+BB VS.AA | HB | 5 | 0.602 | 8.757E-01 | 0.991 (0.834-1.177) | 0.986 (0.831-1.171) |
|  | BA+BB VS.AA | PB | 5 | 0.000 | 3.420E-01 | 1.206 (0.819-1.776) | 1.007 (0.894-1.134) |
|  | BA+BB VS.AA | N | 3 | 0.004 | 9.831E-01 | 1.008 (0.474-2.144) | 0.887 (0.748-1.052) |
|  | BA+BB VS.AA | Y | 8 | 0.000 | 2.678E-01 | 1.153 (0.896-1.484) | 1.092 (0.972-1.227) |
|  | BA+BB VS.AA | PCa | 7 | 0.000 | 1.103E-01 | 1.267 (0.948-1.694) | 1.046 (0.942-1.160) |
|  | BA+BB VS.AA | RCC | 3 | 0.220 | 2.398E-01 | 0.823 (0.563-1.203) | 0.842 (0.631-1.122) |
|  | BB VS. AA | Overall | 11 | 0.120 | 4.581E-01 | 0.964 (0.567-1.639) | 0.882 (0.632-1.230) |
|  | BB VS. AA | Asian | 3 | 0.430 | 2.424E-01 | 1.634 (0.269-9.906) | 2.535 (0.533-12.061) |
|  | BB VS. AA | Caucasian | 5 | 0.140 | 8.424E-01 | 1.089 (0.400-2.968) | 1.056 (0.615-1.815) |
|  | BB VS. AA | Mixed | 3 | 0.066 | 6.409E-01 | 0.822 (0.361-1.873) | 0.709 (0.453-1.110) |
|  | BB VS. AA | HB | 5 | 0.576 | 6.184E-01 | 0.835 (0.404-1.724) | 0.845 (0.436-1.639) |
|  | BB VS. AA | PB | 5 | 0.033 | 8.290E-01 | 0.912 (0.395-2.105) | 0.824 (0.550-1.234) |
|  | BB VS. AA | N | 3 | 0.080 | 6.757E-01 | 0.729 (0.166-3.204) | 0.534 (0.301-0.948) |
|  | BB VS. AA | Y | 8 | 0.338 | 4.607E-01 | 1.085 (0.646-1.822) | 1.171 (0.770-1.781) |
|  | BB VS. AA | PCa | 7 | 0.040 | 8.222E-01 | 1.079 (0.554-2.102) | 0.927 (0.650-1.320) |
|  | BB VS. AA | RCC | 3 | 0.563 | 1.801E-01 | 0.615 (0.196-1.929) | 0.478 (0.163-1.406) |
|  | BB VS.BA+AA | Overall | 11 | 0.141 | 4.630E-01 | 0.951 (0.568-1.592) | 0.883 (0.633-1.231) |
|  | BB VS.BA+AA | Asian | 3 | 0.428 | 2.435E-01 | 1.626 (0.268-9.858) | 2.528 (0.532-12.016) |
|  | BB VS.BA+AA | Caucasian | 5 | 0.140 | 7.942E-01 | 1.101 (0.404-2.998) | 1.075 (0.626-1.844) |
|  | BB VS.BA+AA | Mixed | 3 | 0.094 | 5.545E-01 | 0.794 (0.369-1.707) | 0.703 (0.449-1.100) |
|  | BB VS.BA+AA | HB | 5 | 0.583 | 6.281E-01 | 0.831 (0.403-1.713) | 0.849 (0.438-1.647) |
|  | BB VS.BA+AA | PB | 5 | 0.036 | 8.370E-01 | 0.917 (0.402-2.094) | 0.831 (0.556-1.244) |
|  | BB VS.BA+AA | N | 3 | 0.109 | 3.445E-02 | 0.716 (0.184-2.791) | 0.537 (0.302-0.956) |
|  | BB VS.BA+AA | Y | 8 | 0.327 | 4.721E-01 | 1.083 (0.640-1.832) | 1.166 (0.767-1.771) |
|  | BB VS.BA+AA | PCa | 7 | 0.048 | 8.644E-01 | 1.058 (0.552-2.030) | 0.924 (0.649-1.315) |
|  | BB VS.BA+AA | RCC | 3 | 0.587 | 2.029E-01 | 0.630 (0.201-1.972) | 0.496 (0.168-1.460) |
| HIF1α-rs11549467 | B VS. A | Overall | 8 | 0.621 | 1.683E-01 | 1.179 (0.927-1.498) | 1.181 (0.932-1.497) |
|  | B VS. A | Asian | 3 | 0.182 | 8.555E-02 | 1.251 (0.868-1.803) | 1.271 (0.967-1.670) |
|  | B VS. A | Caucasian | 3 | 0.868 | 8.111E-01 | 0.925 (0.479-1.787) | 0.923 (0.479-1.779) |
|  | B VS. A | Mixed | 2 | 0.474 | 9.360E-01 | 0.961 (0.474-1.947) | 0.972 (0.483-1.955) |
|  | B VS. A | HB | 4 | 0.305 | 9.471E-02 | 1.246 (0.910-1.708) | 1.260 (0.961-1.651) |
|  | B VS. A | PB | 3 | 0.882 | 7.621E-01 | 0.926 (0.562-1.526) | 0.926 (0.562-1.525) |
|  | B VS. A | Y | 7 | 0.545 | 1.862E-01 | 1.172 (0.922-1.492) | 1.174 (0.926-1.489) |
|  | B VS. A | PCa | 4 | 0.490 | **4.413E-02^*^** | 1.461 (1.003-2.128) | 1.465 (1.010-2.124) |
|  | B VS. A | RCC | 3 | 0.777 | 7.087E-01 | 1.074 (0.742-1.554) | 1.073 (0.742-1.551) |
|  | BA VS. AA | Overall | 8 | 0.302 | 4.673E-01 | 1.063 (0.792-1.427) | 1.095 (0.857-1.400) |
|  | BA VS. AA | Asian | 3 | 0.075 | 5.683E-01 | 1.149 (0.714-1.849) | 1.198 (0.904-1.589) |
|  | BA VS. AA | Caucasian | 3 | 0.722 | 3.538E-01 | 0.715 (0.351-1.460) | 0.714 (0.351-1.454) |
|  | BA VS. AA | Mixed | 2 | 0.468 | 9.205E-01 | 0.933 (0.453-1.921) | 0.964 (0.473-1.965) |
|  | BA VS. AA | HB | 4 | 0.147 | 2.267E-01 | 1.140 (0.746-1.743) | 1.188 (0.898-1.571) |
|  | BA VS. AA | PB | 3 | 0.653 | 4.148E-01 | 0.807 (0.478-1.361) | 0.805 (0.478-1.356) |
|  | BA VS. AA | Y | 7 | 0.245 | 5.017E-01 | 1.048 (0.768-1.430) | 1.088 (0.851-1.391) |
|  | BA VS. AA | PCa | 4 | 0.500 | 6.302E-02 | 1.423 (0.969-2.090) | 1.433 (0.981-2.095) |
|  | BA VS. AA | RCC | 3 | 0.402 | 9.885E-01 | 1.006 (0.685-1.477) | 1.003 (0.685-1.468) |
|  | BA+BB VS.AA | Overall | 8 | 0.464 | 2.905E-01 | 1.14 (0.891-1.459) | 1.140 (0.894-1.453) |
|  | BA+BB VS.AA | Asian | 3 | 0.119 | 1.351E-01 | 1.204 (0.788-1.839) | 1.238 (0.936-1.638) |
|  | BA+BB VS.AA | Caucasian | 3 | 0.810 | 5.596E-01 | 0.817 (0.411-1.622) | 0.816 (0.412-1.617) |
|  | BA+BB VS.AA | Mixed | 2 | 0.473 | 9.358E-01 | 0.96 (0.473-1.951) | 0.972 (0.482-1.959) |
|  | BA+BB VS.AA | HB | 4 | 0.215 | 1.481E-01 | 1.197 (0.824-1.738) | 1.227 (0.930-1.620) |
|  | BA+BB VS.AA | PB | 3 | 0.800 | 5.743E-01 | 0.864 (0.518-1.442) | 0.864 (0.518-1.440) |
|  | BA+BB VS.AA | Y | 7 | 0.389 | 3.179E-01 | 1.125 (0.869-1.456) | 1.132 (0.887-1.444) |
|  | BA+BB VS.AA | PCa | 4 | 0.501 | 5.165E-02 | 1.450 (0.991-2.123) | 1.454 (0.997-2.119) |
|  | BA+BB VS.AA | RCC | 3 | 0.595 | 8.456E-01 | 1.04 (0.711-1.520) | 1.038 (0.712-1.515) |
|  | BB VS. AA | Overall | 8 | 0.991 | 6.537E-02 | 5.836 (0.396-85.919) | 9.152 (0.869-96.435) |
|  | BB VS. AA | Asian | 3 | 0.792 | 1.469E-01 | 11.381 (0.201-642.945) | 14.842 (0.388-567.980) |
|  | BB VS. AA | Caucasian | 3 | 0.869 | 2.841E-01 | 6.142 (0.073-519.334) | 9.452 (0.155-575.916) |
|  | BB VS. AA | Mixed | 2 | 0.979 | 9.781E-01 | 1.091 (0.002-537.002) | 1.091 (0.002-536.929) |
|  | BB VS. AA | HB | 4 | 0.905 | 1.282E-01 | 9.006 (0.231-351.773) | 12.914 (0.478-348.631) |
|  | BB VS. AA | PB | 3 | 0.824 | 3.024E-01 | 4.848 (0.057-409.623) | 7.877 (0.156-397.881) |
|  | BB VS. AA | Y | 7 | 0.986 | 6.633E-02 | 7.005 (0.415-118.194) | 10.545 (0.853-130.341) |
|  | BB VS. AA | PCa | 4 | 0.959 | 4.648E-01 | 2.805 (0.052-152.181) | 3.782 (0.107-133.904) |
|  | BB VS. AA | RCC | 3 | 0.842 | 2.952E-01 | 5.552 (0.066-469.44) | 8.648 (0.152-491.302) |
|  | BB VS.BA+AA | Overall | 8 | 0.991 | 6.443E-02 | 5.85 (0.397-86.126) | 9.196 (0.876-96.580) |
|  | BB VS.BA+AA | Asian | 3 | 0.788 | 1.463E-01 | 11.359 (0.201-641.677) | 14.840 (0.390-564.877) |
|  | BB VS.BA+AA | Caucasian | 3 | 0.867 | 2.808E-01 | 6.203 (0.073-524.477) | 9.573 (0.158-580.447) |
|  | BB VS.BA+AA | Mixed | 2 | 0.979 | 9.785E-01 | 1.089 (0.002-535.969) | 1.089 (0.002-535.892) |
|  | BB VS.BA+AA | HB | 4 | 0.903 | 1.276E-01 | 9.003 (0.231-351.635) | 12.929 (0.481-347.784) |
|  | BB VS.BA+AA | PB | 3 | 0.821 | 2.990E-01 | 4.889 (0.058-413.071) | 7.967 (0.159-400.115) |
|  | BB VS.BA+AA | Y | 7 | 0.985 | 6.537E-02 | 7.028 (0.417-118.570) | 10.601 (0.861-130.609) |
|  | BB VS.BA+AA | PCa | 4 | 0.961 | 4.709E-01 | 2.769 (0.051-150.27) | 3.723 (0.104-132.646) |
|  | BB VS.BA+AA | RCC | 3 | 0.840 | 2.922E-01 | 5.597 (0.066-473.194) | 8.742 (0.155-493.968) |
| HRAS-rs12628 | B VS. A | Overall | 4 | 0.000 | 1.930E-01 | 1.374 (0.852-2.217) | 1.204 (1.027-1.411) |
|  | B VS. A | Caucasian | 3 | 0.020 | 6.509E-01 | 1.085 (0.762-1.545) | 1.070 (0.904-1.268) |
|  | B VS. A | HB | 2 | 0.000 | 2.697E-01 | 1.782 (0.639-4.971) | 1.317 (1.071-1.618) |
|  | B VS. A | PB | 2 | 0.005 | 7.957E-01 | 1.097 (0.544-2.215) | 1.057 (0.824-1.356) |
|  | B VS. A | N | 2 | 0.100 | 7.536E-01 | 0.934 (0.672-1.296) | 0.970 (0.803-1.172) |
|  | B VS. A | Y | 2 | 0.037 | 2.084E-02 | 2.162 (1.124-4.158) | 2.034 (1.507-2.746) |
|  | BA VS. AA | Overall | 4 | 0.002 | 4.940E-01 | 1.196 (0.716-1.999) | 1.035 (0.836-1.281) |
|  | BA VS. AA | Caucasian | 3 | 0.136 | 2.958E-01 | 0.926 (0.655-1.309) | 0.883 (0.700-1.115) |
|  | BA VS. AA | HB | 2 | 0.000 | 6.140E-01 | 1.353 (0.418-4.374) | 1.030 (0.783-1.356) |
|  | BA VS. AA | PB | 2 | 0.134 | 8.125E-01 | 1.080 (0.641-1.818) | 1.042 (0.743-1.460) |
|  | BA VS. AA | N | 2 | 0.683 | 7.553E-02 | 0.791 (0.612-1.024) | 0.792 (0.612-1.024) |
|  | BA VS. AA | Y | 2 | 0.170 | 1.264E-03 | 1.907 (1.111-3.274) | 1.904 (1.287-2.817) |
|  | BA+BB VS.AA | Overall | 4 | 0.000 | 3.197E-01 | 1.326 (0.761-2.310) | 1.141 (0.930-1.399) |
|  | BA+BB VS.AA | Caucasian | 3 | 0.071 | 9.522E-01 | 1.012 (0.688-1.487) | 0.964 (0.772-1.204) |
|  | BA+BB VS.AA | HB | 2 | 0.000 | 4.383E-01 | 1.600 (0.488-5.247) | 1.199 (0.923-1.556) |
|  | BA+BB VS.AA | PB | 2 | 0.029 | 7.564E-01 | 1.123 (0.539-2.341) | 1.055 (0.761-1.463) |
|  | BA+BB VS.AA | N | 2 | 0.630 | 2.103E-01 | 0.854 (0.667-1.093) | 0.854 (0.667-1.093) |
|  | BA+BB VS.AA | Y | 2 | 0.127 | **3.647E-05^**^** | 2.220 (1.245-3.960) | 2.211 (1.517-3.222) |
|  | BB VS. AA | Overall | 4 | 0.004 | 7.391E-01 | 1.246 (0.341-4.548) | 1.804 (1.200-2.713) |
|  | BB VS. AA | Caucasian | 3 | 0.003 | 9.428E-01 | 1.048 (0.291-3.770) | 1.547 (1.015-2.357) |
|  | BB VS. AA | HB | 2 | 0.133 | 2.576E-03 | 5.765 (0.084-396.741) | 2.198 (1.317-3.668) |
|  | BB VS. AA | PB | 2 | 0.001 | 7.615E-01 | 0.618 (0.028-13.776) | 1.262 (0.639-2.492) |
|  | BB VS. AA | N | 2 | 0.002 | 6.168E-01 | 0.509 (0.036-7.170) | 1.273 (0.783-2.071) |
|  | BB VS. AA | Y | 2 | 0.163 | **5.725E-04^**^** | 7.207 (0.158-328.963) | 4.174 (1.851-9.412) |
|  | BB VS.BA+AA | Overall | 4 | 0.006 | 7.169E-01 | 1.246 (0.379-4.101) | 1.858 (1.253-2.756) |
|  | BB VS.BA+AA | Caucasian | 3 | 0.005 | 8.967E-01 | 1.082 (0.331-3.537) | 1.636 (1.092-2.451) |
|  | BB VS.BA+AA | HB | 2 | 0.176 | **5.642E-04^**^** | 4.887 (0.138-172.923) | 2.396 (1.458-3.938) |
|  | BB VS.BA+AA | PB | 2 | 0.002 | 7.124E-01 | 0.588 (0.035-9.876) | 1.184 (0.616-2.276) |
|  | BB VS.BA+AA | N | 2 | 0.001 | 6.771E-01 | 0.564 (0.038-8.347) | 1.457 (0.912-2.327) |
|  | BB VS.BA+AA | Y | 2 | 0.169 | 2.548E-03 | 5.705 (0.137-237.099) | 3.274 (1.515-7.074) |
| VEGF-rs10434 | B VS. A | Overall | 3 | 0.474 | 1.778E-01 | 1.095 (0.959-1.251) | 1.095 (0.959-1.250) |
|  | BA VS. AA | Overall | 3 | 0.790 | 4.231E-01 | 1.078 (0.897-1.295) | 1.078 (0.897-1.295) |
|  | BA+BB VS.AA | Overall | 3 | 0.604 | 2.801E-01 | 1.102 (0.924-1.313) | 1.102 (0.924-1.313) |
|  | BB VS. AA | Overall | 3 | 0.449 | 1.586E-01 | 1.258 (0.920-1.720) | 1.251 (0.916-1.707) |
|  | BB VS.BA+AA | Overall | 3 | 0.490 | 2.338E-01 | 1.202 (0.893-1.617) | 1.196 (0.891-1.607) |
| VEGF-rs1570360 | B VS. A | Overall | 8 | 0.006 | 3.373E-01 | 0.935 (0.815-1.073) | 0.956 (0.887-1.031) |
|  | B VS. A | Asian | 2 | 0.238 | 1.964E-01 | 1.099 (0.924-1.307) | 1.101 (0.951-1.275) |
|  | B VS. A | Caucasian | 6 | 0.018 | 1.160E-01 | 0.877 (0.745-1.033) | 0.909 (0.833-0.993) |
|  | B VS. A | HB | 4 | 0.002 | 4.344E-01 | 0.908 (0.714-1.156) | 0.955 (0.865-1.055) |
|  | B VS. A | PB | 4 | 0.146 | 4.691E-01 | 0.950 (0.801-1.126) | 0.958 (0.854-1.076) |
|  | B VS. A | Y | 7 | 0.003 | 3.062E-01 | 0.920 (0.784-1.079) | 0.951 (0.877-1.030) |
|  | B VS. A | BCa | 2 | 0.023 | 8.530E-01 | 1.027 (0.777-1.356) | 0.989 (0.881-1.110) |
|  | B VS. A | PCa | 3 | 0.025 | 8.595E-02 | 0.769 (0.569-1.038) | 0.853 (0.748-0.974) |
|  | B VS. A | RCC | 3 | 0.263 | 5.503E-01 | 1.039 (0.865-1.247) | 1.047 (0.901-1.216) |
|  | BA VS. AA | Overall | 8 | 0.135 | 3.183E-01 | 0.949 (0.823-1.095) | 0.948 (0.852-1.053) |
|  | BA VS. AA | Asian | 2 | 0.759 | 5.524E-01 | 1.066 (0.863-1.317) | 1.066 (0.863-1.317) |
|  | BA VS. AA | Caucasian | 6 | 0.094 | 2.893E-01 | 0.903 (0.748-1.090) | 0.911 (0.806-1.029) |
|  | BA VS. AA | HB | 4 | 0.050 | 3.446E-01 | 0.886 (0.690-1.139) | 0.906 (0.788-1.041) |
|  | BA VS. AA | PB | 4 | 0.503 | 9.278E-01 | 1.008 (0.856-1.187) | 1.008 (0.856-1.186) |
|  | BA VS. AA | Y | 7 | 0.094 | 4.293E-01 | 0.936 (0.793-1.103) | 0.938 (0.839-1.050) |
|  | BA VS. AA | BCa | 2 | 0.143 | 3.295E-01 | 0.946 (0.738-1.212) | 0.923 (0.786-1.084) |
|  | BA VS. AA | PCa | 3 | 0.055 | 3.468E-01 | 0.838 (0.579-1.211) | 0.908 (0.754-1.092) |
|  | BA VS. AA | RCC | 3 | 0.390 | 6.289E-01 | 1.056 (0.849-1.314) | 1.055 (0.849-1.312) |
|  | BA+BB VS.AA | Overall | 8 | 0.028 | 3.942E-01 | 0.932 (0.792-1.096) | 0.944 (0.854-1.043) |
|  | BA+BB VS.AA | Asian | 2 | 0.468 | 3.296E-01 | 1.103 (0.906-1.342) | 1.103 (0.906-1.342) |
|  | BA+BB VS.AA | Caucasian | 6 | 0.036 | 1.753E-01 | 0.869 (0.710-1.064) | 0.894 (0.796-1.004) |
|  | BA+BB VS.AA | HB | 4 | 0.008 | 3.679E-01 | 0.874 (0.653-1.171) | 0.919 (0.806-1.048) |
|  | BA+BB VS.AA | PB | 4 | 0.332 | 7.815E-01 | 0.979 (0.826-1.160) | 0.978 (0.839-1.141) |
|  | BA+BB VS.AA | Y | 7 | 0.017 | 3.506E-01 | 0.915 (0.758-1.103) | 0.935 (0.841-1.040) |
|  | BA+BB VS.AA | BCa | 2 | 0.049 | 9.344E-01 | 0.987 (0.719-1.354) | 0.949 (0.815-1.105) |
|  | BA+BB VS.AA | PCa | 3 | 0.031 | 1.751E-01 | 0.767 (0.522-1.126) | 0.859 (0.721-1.023) |
|  | BA+BB VS.AA | RCC | 3 | 0.280 | 5.695E-01 | 1.050 (0.827-1.333) | 1.061 (0.866-1.300) |
|  | BB VS. AA | Overall | 8 | 0.017 | 3.874E-01 | 0.883 (0.665-1.171) | 0.931 (0.789-1.099) |
|  | BB VS. AA | Asian | 2 | 0.206 | 2.131E-01 | 1.206 (0.820-1.773) | 1.212 (0.896-1.639) |
|  | BB VS. AA | Caucasian | 6 | 0.041 | 1.359E-01 | 0.770 (0.546-1.086) | 0.832 (0.683-1.014) |
|  | BB VS. AA | HB | 4 | 0.018 | 6.348E-01 | 0.898 (0.577-1.399) | 0.972 (0.781-1.210) |
|  | BB VS. AA | PB | 4 | 0.081 | 4.253E-01 | 0.840 (0.547-1.290) | 0.880 (0.684-1.133) |
|  | BB VS. AA | Y | 7 | 0.009 | 3.538E-01 | 0.853 (0.611-1.193) | 0.923 (0.772-1.104) |
|  | BB VS. AA | BCa | 2 | 0.054 | 7.131E-01 | 1.102 (0.656-1.850) | 1.060 (0.816-1.378) |
|  | BB VS. AA | PCa | 3 | 0.041 | 7.173E-02 | 0.542 (0.278-1.056) | 0.688 (0.514-0.923) |
|  | BB VS. AA | RCC | 3 | 0.442 | 6.147E-01 | 1.086 (0.794-1.485) | 1.083 (0.794-1.477) |
|  | BB VS.BA+AA | Overall | 8 | 0.064 | 4.893E-01 | 0.920 (0.726-1.165) | 0.950 (0.812-1.113) |
|  | BB VS.BA+AA | Asian | 2 | 0.214 | 2.707E-01 | 1.170 (0.818-1.674) | 1.174 (0.882-1.562) |
|  | BB VS.BA+AA | Caucasian | 6 | 0.109 | 1.359E-01 | 0.826 (0.620-1.099) | 0.866 (0.717-1.046) |
|  | BB VS.BA+AA | HB | 4 | 0.096 | 9.011E-01 | 0.979 (0.703-1.364) | 1.010 (0.819-1.244) |
|  | BB VS.BA+AA | PB | 4 | 0.097 | 3.884E-01 | 0.840 (0.566-1.248) | 0.877 (0.689-1.114) |
|  | BB VS.BA+AA | Y | 7 | 0.038 | 4.611E-01 | 0.899 (0.678-1.193) | 0.947 (0.798-1.123) |
|  | BB VS.BA+AA | BCa | 2 | 0.121 | 4.764E-01 | 1.121 (0.753-1.670) | 1.096 (0.852-1.408) |
|  | BB VS.BA+AA | PCa | 3 | 0.074 | 8.456E-02 | 0.604 (0.340-1.071) | 0.721 (0.545-0.953) |
|  | BB VS.BA+AA | RCC | 3 | 0.648 | 7.294E-01 | 1.054 (0.785-1.415) | 1.053 (0.786-1.410) |
| VEGF-rs2010963 | B VS. A | Overall | 8 | 0.056 | 9.938E-02 | 1.093 (0.983-1.214) | 1.092 (1.019-1.170) |
|  | B VS. A | Asian | 4 | 0.083 | 2.183E-01 | 1.087 (0.952-1.243) | 1.112 (1.020-1.212) |
|  | B VS. A | Caucasian | 4 | 0.085 | 2.610E-01 | 1.131 (0.913-1.401) | 1.058 (0.943-1.187) |
|  | B VS. A | HB | 6 | 0.060 | 1.141E-01 | 1.095 (0.979-1.224) | 1.096 (1.020-1.178) |
|  | B VS. A | PB | 2 | 0.082 | 5.842E-01 | 1.136 (0.720-1.790) | 1.051 (0.833-1.326) |
|  | B VS. A | RCC | 6 | 0.078 | 1.898E-01 | 1.086 (0.960-1.227) | 1.104 (1.018-1.197) |
|  | B VS. A | other (BCa-PCa) | 2 | 0.058 | 3.832E-01 | 1.193 (0.802-1.773) | 1.061 (0.929-1.211) |
|  | BA VS. AA | Overall | 8 | 0.278 | 2.171E-02 | 1.134 (1.001-1.285) | 1.132 (1.018-1.259) |
|  | BA VS. AA | Asian | 4 | 0.411 | 2.079E-02 | 1.178 (1.025-1.354) | 1.178 (1.025-1.354) |
|  | BA VS. AA | Caucasian | 4 | 0.171 | 4.102E-01 | 1.152 (0.885-1.500) | 1.071 (0.910-1.261) |
|  | BA VS. AA | HB | 6 | 0.187 | 2.699E-02 | 1.138 (0.982-1.318) | 1.134 (1.014-1.269) |
|  | BA VS. AA | PB | 2 | 0.284 | 5.318E-01 | 1.127 (0.775-1.639) | 1.111 (0.798-1.547) |
|  | BA VS. AA | RCC | 6 | 0.530 | **1.762E-02**^*^ | 1.168 (1.027-1.327) | 1.168 (1.027-1.328) |
|  | BA VS. AA | other (BCa-PCa) | 2 | 0.051 | 4.301E-01 | 1.278 (0.695-2.351) | 1.058 (0.877-1.277) |
|  | BA+BB VS.AA | Overall | 8 | 0.100 | 7.599E-03 | 1.148 (0.993-1.327) | 1.146 (1.037-1.266) |
|  | BA+BB VS.AA | Asian | 4 | 0.225 | 6.998E-03 | 1.170 (0.990-1.382) | 1.197 (1.050-1.365) |
|  | BA+BB VS.AA | Caucasian | 4 | 0.086 | 2.428E-01 | 1.193 (0.887-1.603) | 1.077 (0.922-1.258) |
|  | BA+BB VS.AA | HB | 6 | 0.081 | 8.348E-02 | 1.154 (0.981-1.357) | 1.152 (1.037-1.280) |
|  | BA+BB VS.AA | PB | 2 | 0.147 | 5.722E-01 | 1.183 (0.695-2.013) | 1.094 (0.800-1.497) |
|  | BA+BB VS.AA | RCC | 6 | 0.240 | **6.790E-03**^*^ | 1.158 (0.996-1.346) | 1.181 (1.047-1.333) |
|  | BA+BB VS.AA | other (BCa-PCa) | 2 | 0.034 | 3.957E-01 | 1.319 (0.696-2.498) | 1.071 (0.896-1.281) |
|  | BB VS. AA | Overall | 8 | 0.140 | 2.555E-02 | 1.170 (0.959-1.428) | 1.182 (1.021-1.369) |
|  | BB VS. AA | Asian | 4 | 0.162 | 3.314E-02 | 1.172 (0.919-1.494) | 1.214 (1.016-1.452) |
|  | BB VS. AA | Caucasian | 4 | 0.133 | 3.989E-01 | 1.239 (0.799-1.921) | 1.118 (0.863-1.448) |
|  | BB VS. AA | HB | 6 | 0.176 | 2.308E-02 | 1.180 (0.966-1.441) | 1.194 (1.025-1.392) |
|  | BB VS. AA | PB | 2 | 0.075 | 6.611E-01 | 1.258 (0.451-3.507) | 1.050 (0.624-1.768) |
|  | BB VS. AA | RCC | 6 | 0.131 | **3.784E-02**^*^ | 1.156 (0.910-1.468) | 1.196 (1.010-1.416) |
|  | BB VS. AA | other (BCa-PCa) | 2 | 0.122 | 3.870E-01 | 1.361 (0.666-2.780) | 1.141 (0.846-1.538) |
|  | BB VS.BA+AA | Overall | 8 | 0.476 | 2.309E-01 | 1.083 (0.953-1.230) | 1.081 (0.952-1.228) |
|  | BB VS.BA+AA | Asian | 4 | 0.315 | 2.938E-01 | 1.081 (0.917-1.273) | 1.083 (0.933-1.257) |
|  | BB VS.BA+AA | Caucasian | 4 | 0.390 | 5.620E-01 | 1.080 (0.844-1.382) | 1.075 (0.841-1.374) |
|  | BB VS.BA+AA | HB | 6 | 0.507 | 2.140E-01 | 1.088 (0.953-1.241) | 1.087 (0.953-1.241) |
|  | BB VS.BA+AA | PB | 2 | 0.138 | 9.929E-01 | 1.108 (0.509-2.412) | 0.998 (0.610-1.633) |
|  | BB VS.BA+AA | RCC | 6 | 0.327 | 3.163E-01 | 1.074 (0.917-1.259) | 1.076 (0.933-1.241) |
|  | BB VS.BA+AA | other (BCa-PCa) | 2 | 0.388 | 5.010E-01 | 1.101 (0.829-1.462) | 1.102 (0.830-1.463) |
| VEGF-rs3025039 | B VS. A | Overall | 10 | 0.000 | 6.628E-01 | 1.044 (0.859-1.270) | 1.013 (0.938-1.095) |
|  | B VS. A | Asian | 6 | 0.337 | **4.545E-04^**^** | 1.179 (1.067-1.303) | 1.180 (1.076-1.294) |
|  | B VS. A | Caucasian | 4 | 0.017 | 3.711E-01 | 0.852 (0.600-1.210) | 0.710 (0.616-0.820) |
|  | B VS. A | HB | 8 | 0.000 | 7.655E-01 | 1.034 (0.830-1.288) | 1.012 (0.935-1.097) |
|  | B VS. A | PB | 2 | 0.144 | 8.747E-01 | 1.097 (0.676-1.779) | 1.025 (0.757-1.387) |
|  | B VS. A | N | 3 | 0.000 | 8.274E-01 | 0.952 (0.614-1.478) | 0.887 (0.793-0.992) |
|  | B VS. A | Y | 7 | 0.125 | 1.260E-02 | 1.124 (0.966-1.307) | 1.146 (1.030-1.275) |
|  | B VS. A | BCa | 3 | 0.000 | 6.741E-01 | 0.918 (0.615-1.369) | 0.860 (0.769-0.962) |
|  | B VS. A | RCC | 6 | 0.195 | **1.199E-03^*^** | 1.183 (1.028-1.363) | 1.198 (1.074-1.336) |
|  | BA VS. AA | Overall | 10 | 0.000 | 9.481E-01 | 0.991 (0.763-1.288) | 0.895 (0.809-0.989) |
|  | BA VS. AA | Asian | 6 | 0.281 | 7.137E-02 | 1.126 (0.972-1.304) | 1.126 (0.990-1.280) |
|  | BA VS. AA | Caucasian | 4 | 0.001 | 4.407E-01 | 0.822 (0.499-1.353) | 0.625 (0.532-0.736) |
|  | BA VS. AA | HB | 8 | 0.000 | 7.518E-01 | 0.953 (0.707-1.284) | 0.877 (0.790-0.974) |
|  | BA VS. AA | PB | 2 | 0.270 | 5.200E-01 | 1.146 (0.768-1.710) | 1.121 (0.792-1.587) |
|  | BA VS. AA | N | 3 | 0.000 | 6.885E-01 | 0.884 (0.484-1.614) | 0.725 (0.626-0.839) |
|  | BA VS. AA | Y | 7 | 0.153 | 2.550E-01 | 1.075 (0.892-1.295) | 1.084 (0.944-1.245) |
|  | BA VS. AA | BCa | 3 | 0.000 | 5.099E-01 | 0.840 (0.501-1.410) | 0.753 (0.659-0.860) |
|  | BA VS. AA | RCC | 6 | 0.244 | 5.641E-02 | 1.160 (0.961-1.401) | 1.167 (0.996-1.367) |
|  | BA+BB VS.AA | Overall | 10 | 0.000 | 8.342E-01 | 1.027 (0.799-1.322) | 0.948 (0.863-1.041) |
|  | BA+BB VS.AA | Asian | 6 | 0.202 | 7.481E-03 | 1.174 (1.016-1.357) | 1.173 (1.044-1.319) |
|  | BA+BB VS.AA | Caucasian | 4 | 0.003 | 4.216E-01 | 0.829 (0.525-1.310) | 0.644 (0.549-0.755) |
|  | BA+BB VS.AA | HB | 8 | 0.000 | 9.970E-01 | 1.001 (0.751-1.334) | 0.938 (0.851-1.034) |
|  | BA+BB VS.AA | PB | 2 | 0.188 | 6.621E-01 | 1.142 (0.699-1.865) | 1.079 (0.768-1.517) |
|  | BA+BB VS.AA | N | 3 | 0.000 | 7.554E-01 | 0.914 (0.519-1.608) | 0.797 (0.697-0.910) |
|  | BA+BB VS.AA | Y | 7 | 0.089 | 2.889E-01 | 1.112 (0.914-1.351) | 1.126 (0.986-1.286) |
|  | BA+BB VS.AA | BCa | 3 | 0.000 | 5.862E-01 | 0.870 (0.527-1.436) | 0.788 (0.693-0.895) |
|  | BA+BB VS.AA | RCC | 6 | 0.148 | **9.705E-03^*^** | 1.194 (0.986-1.446) | 1.205 (1.046-1.389) |
|  | BB VS. AA | Overall | 10 | 0.639 | 8.347E-04 | 1.382 (1.144-1.669) | 1.375 (1.141-1.658) |
|  | BB VS. AA | Asian | 6 | 0.548 | **7.532E-04^**^** | 1.400 (1.149-1.705) | 1.401 (1.152-1.705) |
|  | BB VS. AA | Caucasian | 4 | 0.422 | 6.583E-01 | 1.208 (0.637-2.288) | 1.149 (0.621-2.126) |
|  | BB VS. AA | HB | 8 | 0.773 | **4.316E-04^**^** | 1.404 (1.160-1.698) | 1.405 (1.163-1.699) |
|  | BB VS. AA | PB | 2 | 0.198 | 4.029E-01 | 0.749 (0.122-4.600) | 0.574 (0.157-2.106) |
|  | BB VS. AA | N | 3 | 0.901 | 9.154E-02 | 1.246 (0.964-1.610) | 1.247 (0.965-1.611) |
|  | BB VS. AA | Y | 7 | 0.491 | 2.022E-03 | 1.561 (1.182-2.061) | 1.540 (1.171-2.027) |
|  | BB VS. AA | BCa | 3 | 0.897 | 1.095E-01 | 1.344 (0.932-1.939) | 1.347 (0.935-1.940) |
|  | BB VS. AA | RCC | 6 | 0.244 | **3.699E-03^*^** | 1.394 (1.043-1.863) | 1.383 (1.111-1.721) |
|  | BB VS.BA+AA | Overall | 10 | 0.797 | 3.345E-03 | 1.310 (1.095-1.568) | 1.306 (1.093-1.560) |
|  | BB VS.BA+AA | Asian | 6 | 0.820 | 4.793E-03 | 1.306 (1.084-1.575) | 1.307 (1.085-1.575) |
|  | BB VS.BA+AA | Caucasian | 4 | 0.360 | 4.177E-01 | 1.317 (0.651-2.665) | 1.287 (0.699-2.369) |
|  | BB VS.BA+AA | HB | 8 | 0.908 | 1.894E-03 | 1.329 (1.109-1.592) | 1.331 (1.111-1.593) |
|  | BB VS.BA+AA | PB | 2 | 0.232 | 3.823E-01 | 0.687 (0.131-3.606) | 0.561 (0.153-2.053) |
|  | BB VS.BA+AA | N | 3 | 0.657 | 1.096E-01 | 1.225 (0.953-1.575) | 1.227 (0.955-1.576) |
|  | BB VS.BA+AA | Y | 7 | 0.675 | 1.050E-02 | 1.404 (1.088-1.813) | 1.392 (1.080-1.793) |
|  | BB VS.BA+AA | BCa | 3 | 0.778 | 8.157E-02 | 1.375 (0.956-1.976) | 1.378 (0.961-1.977) |
|  | BB VS.BA+AA | RCC | 6 | 0.455 | **1.930E-02^*^** | 1.286 (1.046-1.582) | 1.278 (1.041-1.570) |
| VEGF-rs699947 | B VS. A | Overall | 12 | 0.014 | 1.260E-02 | 1.150 (1.030-1.283) | 1.145 (1.069-1.226) |
|  | B VS. A | Asian | 6 | 0.286 | **9.201E-07^**^** | 1.273 (1.139-1.424) | 1.277 (1.158-1.408) |
|  | B VS. A | Caucasian | 5 | 0.098 | 6.110E-01 | 1.047 (0.876-1.251) | 1.042 (0.942-1.154) |
|  | B VS. A | HB | 9 | 0.016 | 3.806E-02 | 1.145 (1.007-1.301) | 1.144 (1.062-1.233) |
|  | B VS. A | PB | 2 | 0.374 | 6.420E-01 | 1.046 (0.866-1.264) | 1.046 (0.866-1.262) |
|  | B VS. A | N | 2 | 0.491 | 4.227E-03 | 1.249 (1.073-1.453) | 1.248 (1.072-1.453) |
|  | B VS. A | Y | 10 | 0.010 | 6.723E-02 | 1.131 (0.991-1.291) | 1.120 (1.038-1.209) |
|  | B VS. A | BCa | 4 | 0.446 | 3.841E-01 | 1.049 (0.942-1.168) | 1.049 (0.942-1.168) |
|  | B VS. A | PCa | 3 | 0.102 | 5.132E-01 | 0.916 (0.687-1.222) | 0.940 (0.782-1.131) |
|  | B VS. A | RCC | 5 | 0.603 | **1.311E-07^**^** | 1.311 (1.186-1.451) | 1.312 (1.186-1.450) |
|  | BA VS. AA | Overall | 12 | 0.010 | 7.316E-02 | 1.173 (0.985-1.397) | 1.160 (1.044-1.289) |
|  | BA VS. AA | Asian | 6 | 0.102 | **6.730E-05^**^** | 1.395 (1.122-1.734) | 1.352 (1.166-1.568) |
|  | BA VS. AA | Caucasian | 5 | 0.152 | 8.140E-01 | 0.995 (0.769-1.289) | 1.020 (0.864-1.205) |
|  | BA VS. AA | HB | 9 | 0.106 | 1.502E-02 | 1.150 (0.975-1.357) | 1.156 (1.029-1.299) |
|  | BA VS. AA | PB | 2 | 0.319 | 8.271E-01 | 0.971 (0.736-1.280) | 0.970 (0.737-1.277) |
|  | BA VS. AA | N | 2 | 0.013 | 7.390E-01 | 0.894 (0.461-1.732) | 1.042 (0.832-1.304) |
|  | BA VS. AA | Y | 10 | 0.040 | 2.560E-02 | 1.236 (1.026-1.488) | 1.196 (1.061-1.348) |
|  | BA VS. AA | BCa | 4 | 0.188 | 6.065E-02 | 1.259 (0.930-1.704) | 1.189 (0.992-1.425) |
|  | BA VS. AA | PCa | 3 | 0.461 | 7.603E-02 | 0.792 (0.614-1.023) | 0.793 (0.614-1.025) |
|  | BA VS. AA | RCC | 5 | 0.109 | **5.669E-04^**^** | 1.347 (1.081-1.678) | 1.306 (1.122-1.521) |
|  | BA+BB VS.AA | Overall | 12 | 0.019 | 1.578E-02 | 1.213 (1.037-1.418) | 1.201 (1.088-1.326) |
|  | BA+BB VS.AA | Asian | 6 | 0.221 | **1.339E-06^**^** | 1.427 (1.198-1.700) | 1.410 (1.226-1.620) |
|  | BA+BB VS.AA | Caucasian | 5 | 0.294 | 5.653E-01 | 1.040 (0.857-1.262) | 1.047 (0.896-1.222) |
|  | BA+BB VS.AA | HB | 9 | 0.146 | 9.585E-04 | 1.200 (1.036-1.390) | 1.202 (1.078-1.340) |
|  | BA+BB VS.AA | PB | 2 | 0.304 | 9.627E-01 | 1.008 (0.770-1.318) | 1.006 (0.776-1.304) |
|  | BA+BB VS.AA | N | 2 | 0.097 | 5.921E-01 | 1.111 (0.755-1.636) | 1.183 (0.963-1.454) |
|  | BA+BB VS.AA | Y | 10 | 0.018 | 2.432E-02 | 1.241 (1.028-1.497) | 1.207 (1.078-1.351) |
|  | BA+BB VS.AA | BCa | 4 | 0.243 | 9.641E-02 | 1.207 (0.932-1.561) | 1.156 (0.974-1.371) |
|  | BA+BB VS.AA | PCa | 3 | 0.837 | 2.077E-01 | 0.859 (0.679-1.087) | 0.859 (0.679-1.088) |
|  | BA+BB VS.AA | RCC | 5 | 0.189 | **4.602E-06^**^** | 1.417 (1.179-1.703) | 1.394 (1.209-1.607) |
|  | BB VS. AA | Overall | 12 | 0.022 | 1.276E-02 | 1.336 (1.064-1.679) | 1.300 (1.129-1.497) |
|  | BB VS. AA | Asian | 6 | 0.201 | **1.706E-05^**^** | 1.540 (1.185-2.003) | 1.572 (1.279-1.931) |
|  | BB VS. AA | Caucasian | 5 | 0.042 | 5.051E-01 | 1.156 (0.755-1.77) | 1.103 (0.901-1.350) |
|  | BB VS. AA | HB | 9 | 0.010 | 7.330E-02 | 1.296 (0.976-1.720) | 1.279 (1.097-1.491) |
|  | BB VS. AA | PB | 2 | 0.719 | 3.481E-01 | 1.218 (0.804-1.847) | 1.219 (0.806-1.845) |
|  | BB VS. AA | N | 2 | 0.788 | 2.685E-03 | 1.594 (1.177-2.159) | 1.592 (1.175-2.157) |
|  | BB VS. AA | Y | 10 | 0.015 | 9.756E-02 | 1.269 (0.957-1.681) | 1.230 (1.049-1.443) |
|  | BB VS. AA | BCa | 4 | 0.424 | 6.808E-01 | 1.048 (0.838-1.310) | 1.048 (0.839-1.309) |
|  | BB VS. AA | PCa | 3 | 0.020 | 6.809E-01 | 0.822 (0.322-2.096) | 1.009 (0.680-1.498) |
|  | BB VS. AA | RCC | 5 | 0.772 | **7.220E-07^**^** | 1.688 (1.372-2.076) | 1.687 (1.372-2.075) |
|  | BB VS.BA+AA | Overall | 12 | 0.021 | 7.952E-02 | 1.194 (0.979-1.456) | 1.166 (1.031-1.318) |
|  | BB VS.BA+AA | Asian | 6 | 0.084 | 1.014E-01 | 1.243 (0.958-1.613) | 1.286 (1.074-1.540) |
|  | BB VS.BA+AA | Caucasian | 5 | 0.027 | 5.619E-01 | 1.124 (0.758-1.665) | 1.066 (0.897-1.268) |
|  | BB VS.BA+AA | HB | 9 | 0.004 | 1.889E-01 | 1.189 (0.918-1.540) | 1.165 (1.019-1.331) |
|  | BB VS.BA+AA | PB | 2 | 0.959 | 3.950E-01 | 1.173 (0.812-1.696) | 1.173 (0.812-1.696) |
|  | BB VS.BA+AA | N | 2 | 0.568 | 1.695E-03 | 1.571 (1.181-2.089) | 1.576 (1.186-2.094) |
|  | BB VS.BA+AA | Y | 10 | 0.042 | 3.540E-01 | 1.110 (0.890-1.383) | 1.088 (0.949-1.247) |
|  | BB VS.BA+AA | BCa | 4 | 0.408 | 7.653E-01 | 0.974 (0.813-1.167) | 0.973 (0.813-1.165) |
|  | BB VS.BA+AA | PCa | 3 | 0.009 | 8.059E-01 | 0.882 (0.325-2.395) | 1.140 (0.784-1.658) |
|  | BB VS.BA+AA | RCC | 5 | 0.780 | **1.791E-04^**^** | 1.436 (1.189-1.735) | 1.435 (1.188-1.733) |
| VEGF-rs833061 | B VS. A | Overall | 8 | 0.000 | 7.069E-01 | 0.968 (0.815-1.148) | 1.047 (0.969-1.131) |
|  | B VS. A | Asian | 4 | 0.001 | 8.182E-01 | 0.968 (0.733-1.279) | 1.073 (0.960-1.200) |
|  | B VS. A | Caucasian | 3 | 0.004 | 4.462E-01 | 0.872 (0.614-1.240) | 1.008 (0.900-1.129) |
|  | B VS. A | HB | 5 | 0.003 | 9.247E-01 | 1.009 (0.837-1.217) | 1.067 (0.980-1.162) |
|  | B VS. A | PB | 3 | 0.004 | 5.437E-01 | 0.866 (0.544-1.379) | 0.959 (0.799-1.152) |
|  | B VS. A | N | 3 | 0.001 | 8.623E-01 | 0.958 (0.591-1.552) | 1.146 (0.987-1.329) |
|  | B VS. A | Y | 5 | 0.024 | 6.726E-01 | 0.964 (0.812-1.144) | 1.013 (0.925-1.108) |
|  | B VS. A | BCa | 2 | 0.880 | 3.601E-01 | 1.051 (0.945-1.169) | 1.051 (0.945-1.169) |
|  | B VS. A | PCa | 3 | 0.014 | 4.762E-01 | 0.862 (0.574-1.296) | 0.916 (0.759-1.105) |
|  | B VS. A | RCC | 3 | 0.000 | 7.202E-01 | 0.920 (0.581-1.455) | 1.120 (0.974-1.287) |
|  | BA VS. AA | Overall | 8 | 0.003 | 7.511E-01 | 0.964 (0.769-1.209) | 1.021 (0.907-1.150) |
|  | BA VS. AA | Asian | 4 | 0.000 | 4.835E-01 | 0.861 (0.568-1.307) | 0.998 (0.852-1.170) |
|  | BA VS. AA | Caucasian | 3 | 0.414 | 9.260E-01 | 1.008 (0.834-1.219) | 1.009 (0.835-1.220) |
|  | BA VS. AA | HB | 5 | 0.001 | 5.685E-01 | 0.918 (0.686-1.230) | 1.009 (0.887-1.148) |
|  | BA VS. AA | PB | 3 | 0.200 | 5.729E-01 | 1.065 (0.723-1.568) | 1.089 (0.810-1.465) |
|  | BA VS. AA | N | 3 | 0.000 | 6.895E-01 | 0.846 (0.372-1.923) | 1.003 (0.795-1.266) |
|  | BA VS. AA | Y | 5 | 0.729 | 6.974E-01 | 1.027 (0.895-1.179) | 1.028 (0.896-1.179) |
|  | BA VS. AA | BCa | 2 | 0.777 | 5.352E-01 | 1.053 (0.894-1.240) | 1.053 (0.894-1.240) |
|  | BA VS. AA | PCa | 3 | 0.000 | 5.247E-01 | 0.776 (0.355-1.695) | 0.842 (0.649-1.093) |
|  | BA VS. AA | RCC | 3 | 0.235 | 3.434E-01 | 1.079 (0.802-1.451) | 1.117 (0.888-1.406) |
|  | BA+BB VS.AA | Overall | 8 | 0.000 | 7.122E-01 | 0.957 (0.758-1.209) | 1.050 (0.942-1.171) |
|  | BA+BB VS.AA | Asian | 4 | 0.000 | 5.690E-01 | 0.891 (0.599-1.325) | 1.050 (0.910-1.211) |
|  | BA+BB VS.AA | Caucasian | 3 | 0.069 | 6.616E-01 | 0.920 (0.634-1.335) | 1.013 (0.847-1.212) |
|  | BA+BB VS.AA | HB | 5 | 0.001 | 7.118E-01 | 0.949 (0.717-1.255) | 1.052 (0.935-1.184) |
|  | BA+BB VS.AA | PB | 3 | 0.033 | 8.653E-01 | 0.953 (0.549-1.656) | 1.037 (0.779-1.379) |
|  | BA+BB VS.AA | N | 3 | 0.000 | 7.568E-01 | 0.882 (0.399-1.951) | 1.111 (0.909-1.357) |
|  | BA+BB VS.AA | Y | 5 | 0.233 | 7.049E-01 | 1.008 (0.855-1.188) | 1.025 (0.900-1.168) |
|  | BA+BB VS.AA | BCa | 2 | 0.952 | 4.399E-01 | 1.063 (0.911-1.239) | 1.063 (0.911-1.239) |
|  | BA+BB VS.AA | PCa | 3 | 0.001 | 5.257E-01 | 0.788 (0.378-1.643) | 0.854 (0.665-1.096) |
|  | BA+BB VS.AA | RCC | 3 | 0.017 | 9.278E-01 | 0.978 (0.607-1.575) | 1.171 (0.962-1.426) |
|  | BB VS. AA | Overall | 8 | 0.104 | 1.951E-01 | 1.100 (0.862-1.405) | 1.111 (0.948-1.301) |
|  | BB VS. AA | Asian | 4 | 0.415 | 1.065E-01 | 1.209 (0.963-1.519) | 1.206 (0.961-1.515) |
|  | BB VS. AA | Caucasian | 3 | 0.013 | 6.241E-01 | 0.832 (0.400-1.733) | 1.015 (0.808-1.274) |
|  | BB VS. AA | HB | 5 | 0.550 | 7.792E-02 | 1.169 (0.984-1.389) | 1.168 (0.983-1.387) |
|  | BB VS. AA | PB | 3 | 0.010 | 4.869E-01 | 0.645 (0.187-2.220) | 0.842 (0.560-1.266) |
|  | BB VS. AA | N | 3 | 0.645 | 2.723E-02 | 1.379 (1.038-1.832) | 1.378 (1.037-1.831) |
|  | BB VS. AA | Y | 5 | 0.068 | 9.542E-01 | 0.990 (0.703-1.394) | 1.010 (0.834-1.222) |
|  | BB VS. AA | BCa | 2 | 0.701 | 4.566E-01 | 1.089 (0.870-1.362) | 1.089 (0.870-1.362) |
|  | BB VS. AA | PCa | 3 | 0.778 | 9.720E-01 | 1.009 (0.622-1.635) | 1.009 (0.622-1.635) |
|  | BB VS. AA | RCC | 3 | 0.006 | 7.580E-01 | 0.878 (0.383-2.011) | 1.170 (0.908-1.508) |
|  | BB VS.BA+AA | Overall | 8 | 0.203 | 3.195E-01 | 1.088 (0.894-1.324) | 1.075 (0.932-1.240) |
|  | BB VS.BA+AA | Asian | 4 | 0.582 | 1.474E-01 | 1.179 (0.946-1.470) | 1.177 (0.944-1.467) |
|  | BB VS.BA+AA | Caucasian | 3 | 0.025 | 7.150E-01 | 0.898 (0.505-1.598) | 1.009 (0.833-1.222) |
|  | BB VS.BA+AA | HB | 5 | 0.707 | 1.086E-01 | 1.138 (0.973-1.330) | 1.136 (0.972-1.328) |
|  | BB VS.BA+AA | PB | 3 | 0.021 | 4.267E-01 | 0.658 (0.235-1.846) | 0.811 (0.568-1.158) |
|  | BB VS.BA+AA | N | 3 | 0.800 | 5.723E-02 | 1.304 (0.993-1.713) | 1.303 (0.992-1.711) |
|  | BB VS.BA+AA | Y | 5 | 0.111 | 9.852E-01 | 0.994 (0.748-1.321) | 1.002 (0.847-1.184) |
|  | BB VS.BA+AA | BCa | 2 | 0.646 | 4.884E-01 | 1.071 (0.882-1.302) | 1.071 (0.882-1.301) |
|  | BB VS.BA+AA | PCa | 3 | 0.949 | 9.792E-01 | 1.006 (0.635-1.596) | 1.006 (0.635-1.595) |
|  | BB VS.BA+AA | RCC | 3 | 0.011 | 8.302E-01 | 0.928 (0.466-1.845) | 1.100 (0.869-1.391) |

SNP: single nucleotide polymorphism; *P*_H_: *P* value of Q test for heterogeneity test; *P*_A_: *P* < 0.05 was considered as statistically significant (bold font mark*) for cancer type subgroup analysis. And multiple testing *P* value according to Bonferroni correction [*P* < 0.05 / (12 polymorphisms x 5 models)] was considered as statistically significant (bold font mark**); PCa: prostate cancer; RCC: renal cell carcinoma; BCa: bladder cancer; HB: hospital based; PB: population based; HWE: Hardy Weinberg equilibrium. Heterogeneity was considered significant when the *P* value < 0.1. A fixed effects model (Der-Simonian Laird) was used if there was no significant heterogeneity; otherwise, a random effects model (Der-Simonian Laird) was used.

**Supplementary table 4.** Details of the sensitivity analyses for the polymorphisms in VEGF/hypoxia/angiogenesis genes and the risk of urogenital carcinomas

A: wild allele; B: mutated allele.

| **Polymorphism** | **Comparison** | **Study Omitted** | **Estimate** | **[95% Confident Interval]** | **Effect Model** | |
| --- | --- | --- | --- | --- | --- | --- |
| VEGF-rs10434 | B vs. A | Abe A et al. (2002) | 1.120 | 0.976-1.285 | Fixed | |
|  |  | Shen BL et al. (2015) | 1.061 | 0.899-1.252 | | |
|  |  | Lu GJ et al. (2015) | 1.095 | 0.896-1.339 | | |
|  | BA vs. AA | Abe A et al. (2002) | 1.101 | 0.906-1.338 | Fixed | |
|  |  | Shen BL et al. (2015) | 1.049 | 0.834-1.319 | | |
|  |  | Lu GJ et al. (2015) | 1.075 | 0.821-1.406 | | |
|  | BA+BB vs. AA | Abe A et al. (2002) | 1.134 | 0.942-1.366 | Fixed | |
|  |  | Shen BL et al. (2015) | 1.062 | 0.854-1.322 | | |
|  |  | Lu GJ et al. (2015) | 1.095 | 0.846-1.418 | | |
|  | BB vs. AA | Abe A et al. (2002) | 1.292 | 0.943-1.771 | Fixed | |
|  |  | Shen BL et al. (2015) | 1.165 | 0.790-1.718 | | |
|  |  | Lu GJ et al. (2015) | 1.296 | 0.782-2.146 | | |
|  | BB vs. BA+AA | Abe A et al. (2002) | 1.230 | 0.913-1.658 | Fixed | |
|  |  | Shen BL et al. (2015) | 1.127 | 0.781-1.628 | | |
|  |  | Lu GJ et al. (2015) | 1.232 | 0.760-1.997 | | |
| VEGF-rs1570360 | B vs. A | McCarron et al. (2002) | 0.951 | 0.819-1.105 | Random | |
|  |  | Sfar et al. (2006) | 0.982 | 0.881-1.094 | | |
|  |  | Garcia-Closas M et al. (2007) | 0.934 | 0.789-1.106 | | |
|  |  | Jacobs et al. (2008) | 0.926 | 0.782-1.096 | | |
|  |  | Ricketts C et al. (2009) | 0.904 | 0.782-1.044 | | |
|  |  | Bruyre F et al. (2010) | 0.945 | 0.818-1.091 | | |
|  |  | Yang Y et al. (2014) | 0.901 | 0.784-1.034 | | |
|  |  | Xian W et al. (2015) | 0.920 | 0.784-1.079 | | |
|  | BA vs. AA | McCarron et al. (2002) | 0.939 | 0.840-1.048 | | Fixed |
|  |  | Sfar et al. (2006) | 0.971 | 0.872-1.081 | | |
|  |  | Garcia-Closas M et al. (2007) | 0.992 | 0.874-1.125 | | |
|  |  | Jacobs et al. (2008) | 0.947 | 0.840-1.067 | | |
|  |  | Ricketts C et al. (2009) | 0.923 | 0.825-1.032 | | |
|  |  | Bruyre F et al. (2010) | 0.954 | 0.857-1.063 | | |
|  |  | Yang Y et al. (2014) | 0.925 | 0.825-1.037 | | |
|  |  | Xian W et al. (2015) | 0.938 | 0.839-1.050 | | |
|  | BA+BB vs. AA | McCarron et al. (2002) | 0.930 | 0.774-1.118 | | Random |
|  |  | Sfar et al. (2006) | 0.977 | 0.868-1.098 | | |
|  |  | Garcia-Closas M et al. (2007) | 0.944 | 0.778-1.147 | | |
|  |  | Jacobs et al. (2008) | 0.923 | 0.755-1.127 | | |
|  |  | Ricketts C et al. (2009) | 0.897 | 0.758-1.060 | | |
|  |  | Bruyre F et al. (2010) | 0.945 | 0.798-1.120 | | |
|  |  | Yang Y et al. (2014) | 0.896 | 0.754-1.064 | | |
|  |  | Xian W et al. (2015) | 0.915 | 0.758-1.103 | | |
|  | BB vs. AA | McCarron et al. (2002) | 0.961 | 0.735-1.257 | | Random |
|  |  | Sfar et al. (2006) | 0.957 | 0.748-1.223 | | |
|  |  | Garcia-Closas M et al. (2007) | 0.870 | 0.613-1.233 | | |
|  |  | Jacobs et al. (2008) | 0.866 | 0.612-1.226 | | |
|  |  | Ricketts C et al. (2009) | 0.825 | 0.607-1.119 | | |
|  |  | Bruyre F et al. (2010) | 0.891 | 0.660-1.204 | | |
|  |  | Yang Y et al. (2014) | 0.816 | 0.615-1.082 | | |
|  |  | Xian W et al. (2015) | 0.853 | 0.611-1.193 | | |
|  | BB vs. BA+AA | McCarron et al. (2002) | 1.002 | 0.825-1.216 | | Random |
|  |  | Sfar et al. (2006) | 0.965 | 0.774-1.203 | | |
|  |  | Garcia-Closas M et al. (2007) | 0.902 | 0.671-1.211 | | |
|  |  | Jacobs et al. (2008) | 0.909 | 0.679-1.217 | | |
|  |  | Ricketts C et al. (2009) | 0.874 | 0.673-1.136 | | |
|  |  | Bruyre F et al. (2010) | 0.921 | 0.715-1.187 | | |
|  |  | Yang Y et al. (2014) | 0.862 | 0.684-1.087 | | |
|  |  | Xian W et al. (2015) | 0.899 | 0.678-1.193 | | |
| VEGF-rs2010963 | B vs. A | Sfar et al. (2006) | 1.072 | 0.969-1.186 | | Random |
|  |  | Garcia-Closas M et al. (2007) | 1.113 | 0.982-1.260 | | |
|  |  | Bruyre F et al. (2010) | 1.077 | 0.970-1.196 | | |
|  |  | Senz-Lpez P et al. (2013) | 1.112 | 0.994-1.243 | | |
|  |  | Qin C et al. (2014) | 1.058 | 0.949-1.179 | | |
|  |  | Shen BL et al. (2015) | 1.091 | 0.965-1.233 | | |
|  |  | Lu GJ et al. (2015) | 1.098 | 0.967-1.247 | | |
|  |  | Xian W et al. (2015) | 1.123 | 1.016-1.242 | | |
|  | BA vs. AA | Sfar et al. (2006) | 1.114 | 1.000-1.241 | | Fixed |
|  |  | Garcia-Closas M et al. (2007) | 1.192 | 0.052-1.352 | | |
|  |  | Bruyre F et al. (2010) | 1.124 | 1.009-1.251 | | |
|  |  | Senz-Lpez P et al. (2013) | 1.144 | 1.024-1.277 | | |
|  |  | Qin C et al. (2014) | 1.075 | 0.949-1.217 | | |
|  |  | Shen BL et al. (2015) | 1.129 | 1.010-1.263 | | |
|  |  | Lu GJ et al. (2015) | 1.137 | 1.013-1.276 | | |
|  |  | Xian W et al. (2015) | 1.148 | 1.030-1.279 | | |
|  | BA+BB vs. AA | Sfar et al. (2006) | 1.127 | 1.019-1.248 | | Fixed |
|  |  | Garcia-Closas M et al. (2007) | 1.206 | 0.072-1.357 | | |
|  |  | Bruyre F et al. (2010) | 1.135 | 1.026-1.256 | | |
|  |  | Senz-Lpez P et al. (2013) | 1.163 | 1.048-1.291 | | |
|  |  | Qin C et al. (2014) | 1.081 | 0.961-1.215 | | |
|  |  | Shen BL et al. (2015) | 1.143 | 1.028-1.270 | | |
|  |  | Lu GJ et al. (2015) | 1.151 | 1.032-1.284 | | |
|  |  | Xian W et al. (2015) | 1.164 | 1.051-1.290 | | |
|  | BB vs. AA | Sfar et al. (2006) | 1.162 | 1.001-1.349 | | Fixed |
|  |  | Garcia-Closas M et al. (2007) | 1.221 | 0.035-1.442 | | |
|  |  | Bruyre F et al. (2010) | 1.166 | 1.005-1.353 | | |
|  |  | Senz-Lpez P et al. (2013) | 1.211 | 1.041-1.409 | | |
|  |  | Qin C et al. (2014) | 1.090 | 0.916-1.298 | | |
|  |  | Shen BL et al. (2015) | 1.178 | 1.007-1.378 | | |
|  |  | Lu GJ et al. (2015) | 1.190 | 1.011-1.401 | | |
|  |  | Xian W et al. (2015) | 1.231 | 1.056-1.436 | | |
|  | BB vs. BA+AA | Sfar et al. (2006) | 1.072 | 0.942-1.219 | | Fixed |
|  |  | Garcia-Closas M et al. (2007) | 1.087 | 0.944-1.251 | | |
|  |  | Bruyre F et al. (2010) | 1.071 | 0.942-1.218 | | |
|  |  | Senz-Lpez P et al. (2013) | 1.098 | 0.963-1.251 | | |
|  |  | Qin C et al. (2014) | 1.027 | 0.886-1.191 | | |
|  |  | Shen BL et al. (2015) | 1.076 | 0.940-1.232 | | |
|  |  | Lu GJ et al. (2015) | 1.080 | 0.939-1.243 | | |
|  |  | Xian W et al. (2015) | 1.137 | 0.987-1.309 | | |
| VEGF-rs3025039 | B vs. A | Abe A et al. (2002) | 1.055 | 0.856-1.301 | | Random |
|  |  | Sfar et al. (2006) | 1.063 | 0.867-1.304 | | |
|  |  | Garcia-Closas M et al. (2007) | 1.149 | 0.037-1.274 | | |
|  |  | Bruyre F et al. (2010) | 1.020 | 0.834-1.249 | | |
|  |  | Senz-Lpez P et al. (2013) | 1.061 | 0.859-1.310 | | |
|  |  | Wang YH et al. (2013) | 1.042 | 0.831-1.307 | | |
|  |  | Yang Y et al. (2014) | 1.029 | 0.828-1.279 | | |
|  |  | Shen BL et al. (2015) | 1.025 | 0.826-1.272 | | |
|  |  | Lu GJ et al. (2015) | 1.031 | 0.825-1.289 | | |
|  |  | Xian W et al. (2015) | 1.001 | 0.822-1.220 | | |
|  | BA vs. AA | Abe A et al. (2002) | 1.022 | 0.771-1.353 | | Random |
|  |  | Sfar et al. (2006) | 1.021 | 0.775-1.345 | | |
|  |  | Garcia-Closas M et al. (2007) | 1.105 | 0.965-1.265 | | |
|  |  | Bruyre F et al. (2010) | 0.958 | 0.731-1.256 | | |
|  |  | Senz-Lpez P et al. (2013) | 0.991 | 0.744-1.320 | | |
|  |  | Wang YH et al. (2013) | 0.989 | 0.730-1.338 | | |
|  |  | Yang Y et al. (2014) | 0.975 | 0.729-1.304 | | |
|  |  | Shen BL et al. (2015) | 0.971 | 0.731-1.290 | | |
|  |  | Lu GJ et al. (2015) | 0.973 | 0.729-1.299 | | |
|  |  | Xian W et al. (2015) | 0.943 | 0.722-1.230 | | |
|  | BA+BB vs. AA | Abe A et al. (2002) | 1.052 | 0.802-1.378 | | Random |
|  |  | Sfar et al. (2006) | 1.056 | 0.810-1.376 | | |
|  |  | Garcia-Closas M et al. (2007) | 1.141 | 0.996-1.308 | | |
|  |  | Bruyre F et al. (2010) | 0.995 | 0.767-1.291 | | |
|  |  | Senz-Lpez P et al. (2013) | 1.037 | 0.787-1.367 | | |
|  |  | Wang YH et al. (2013) | 1.026 | 0.766-1.374 | | |
|  |  | Yang Y et al. (2014) | 1.011 | 0.764-1.337 | | |
|  |  | Shen BL et al. (2015) | 1.006 | 0.763-1.326 | | |
|  |  | Lu GJ et al. (2015) | 1.011 | 0.762-1.342 | | |
|  |  | Xian W et al. (2015) | 0.971 | 0.755-1.249 | | |
|  | BB vs. AA | Abe A et al. (2002) | 1.370 | 1.133-1.658 | | Fixed |
|  |  | Sfar et al. (2006) | 1.380 | 1.142-1.667 | | |
|  |  | Garcia-Closas M et al. (2007) | 1.379 | 1.135-1.675 | | |
|  |  | Bruyre F et al. (2010) | 1.377 | 1.140-1.664 | | |
|  |  | Senz-Lpez P et al. (2013) | 1.409 | 1.165-1.703 | | |
|  |  | Wang YH et al. (2013) | 1.405 | 1.149-1.719 | | |
|  |  | Yang Y et al. (2014) | 1.374 | 1.128-1.672 | | |
|  |  | Shen BL et al. (2015) | 1.407 | 1.141-1.735 | | |
|  |  | Lu GJ et al. (2015) | 1.469 | 1.175-1.837 | | |
|  |  | Xian W et al. (2015) | 1.264 | 1.022-1.562 | | |
|  | BB vs. BA+AA | Abe A et al. (2002) | 1.290 | 1.078-1.545 | | Fixed |
|  |  | Sfar et al. (2006) | 1.302 | 1.089-1.557 | | |
|  |  | Garcia-Closas M et al. (2007) | 1.285 | 1.070-1.544 | | |
|  |  | Bruyre F et al. (2010) | 1.303 | 1.090-1.557 | | |
|  |  | Senz-Lpez P et al. (2013) | 1.334 | 1.114-1.596 | | |
|  |  | Wang YH et al. (2013) | 1.317 | 1.090-1.592 | | |
|  |  | Yang Y et al. (2014) | 1.297 | 1.077-1.560 | | |
|  |  | Shen BL et al. (2015) | 1.324 | 1.088-1.611 | | |
|  |  | Lu GJ et al. (2015) | 1.370 | 1.112-1.688 | | |
|  |  | Xian W et al. (2015) | 1.241 | 1.009-1.527 | | |
| VEGF-rs699947 | B vs. A | Kim EJ et al. (2005) | 1.166 | 1.040-1.307 | | Random |
|  |  | Garcia-Closas M et al. (2007) | 1.170 | 1.037-1.319 | | |
|  |  | VanCleave TT et al. (2010) | 1.169 | 1.042-1.311 | | |
|  |  | Ajaz S et al. (2011) | 1.127 | 1.011-1.256 | | |
|  |  | Henrquez-Hernndez et al. (2012) | 1.138 | 1.018-1.272 | | |
|  |  | Senz-Lpez P et al. (2013) | 1.151 | 1.020-1.299 | | |
|  |  | Ianni M et al. (2013) | 1.150 | 1.021-1.295 | | |
|  |  | Jaiswal PK et al. (2013) | 1.153 | 1.023-1.300 | | |
|  |  | Martinez-Fierro et al (2013) | 1.181 | 1.073-1.300 | | |
|  |  | Shen BL et al. (2015) | 1.129 | 1.005-1.269 | | |
|  |  | Lu GJ et al. (2015) | 1.132 | 1.004-1.277 | | |
|  |  | Xian W et al. (2015) | 1.127 | 1.005-1.265 | | |
|  | BA vs. AA | Kim EJ et al. (2005) | 1.187 | 0.991-1.421 | | Random |
|  |  | Garcia-Closas M et al. (2007) | 1.190 | 0.969-1.460 | | |
|  |  | VanCleave TT et al. (2010) | 1.213 | 1.011-1.455 | | |
|  |  | Ajaz S et al. (2011) | 1.117 | 0.967-1.291 | | |
|  |  | Henrquez-Hernndez et al. (2012) | 1.157 | 0.969-1.381 | | |
|  |  | Senz-Lpez P et al. (2013) | 1.177 | 0.973-1.424 | | |
|  |  | Ianni M et al. (2013) | 1.228 | 1.048-1.438 | | |
|  |  | Jaiswal PK et al. (2013) | 1.135 | 0.948-1.358 | | |
|  |  | Martinez-Fierro et al (2013) | 1.193 | 0.992-1.434 | | |
|  |  | Shen BL et al. (2015) | 1.165 | 0.958-1.417 | | |
|  |  | Lu GJ et al. (2015) | 1.170 | 0.956-1.431 | | |
|  |  | Xian W et al. (2015) | 1.161 | 0.956-1.409 | | |
|  | BA+BB vs. AA | Kim EJ et al. (2005) | 1.227 | 1.045-1.441 | | Random |
|  |  | Garcia-Closas M et al. (2007) | 1.237 | 1.034-1.479 | | |
|  |  | VanCleave TT et al. (2010) | 1.252 | 1.066-1.471 | | |
|  |  | Ajaz S et al. (2011) | 1.165 | 1.022-1.328 | | |
|  |  | Henrquez-Hernndez et al. (2012) | 1.195 | 1.021-1.399 | | |
|  |  | Senz-Lpez P et al. (2013) | 1.216 | 1.025-1.443 | | |
|  |  | Ianni M et al. (2013) | 1.247 | 1.062-1.465 | | |
|  |  | Jaiswal PK et al. (2013) | 1.192 | 1.008-1.409 | | |
|  |  | Martinez-Fierro et al (2013) | 1.247 | 1.069-1.456 | | |
|  |  | Shen BL et al. (2015) | 1.196 | 1.005-1.422 | | |
|  |  | Lu GJ et al. (2015) | 1.201 | 1.004-1.436 | | |
|  |  | Xian W et al. (2015) | 1.191 | 1.004-1.412 | | |
|  | BB vs.AA | Kim EJ et al. (2005) | 1.371 | 1.083-1.735 | | Random |
|  |  | Garcia-Closas M et al. (2007) | 1.395 | 1.090-1.786 | | |
|  |  | VanCleave TT et al. (2010) | 1.353 | 1.060-1.728 | | |
|  |  | Ajaz S et al. (2011) | 1.290 | 1.022-1.629 | | |
|  |  | Henrquez-Hernndez et al. (2012) | 1.308 | 1.035-1.653 | | |
|  |  | Senz-Lpez P et al. (2013) | 1.336 | 1.038-1.720 | | |
|  |  | Ianni M et al. (2013) | 1.318 | 1.027-1.691 | | |
|  |  | Jaiswal PK et al. (2013) | 1.386 | 1.095-1.755 | | |
|  |  | Martinez-Fierro et al (2013) | 1.390 | 1.153-1.677 | | |
|  |  | Shen BL et al. (2015) | 1.290 | 1.009-1.650 | | |
|  |  | Lu GJ et al.(2015) | 1.294 | 1.003-1.669 | | |
|  |  | Xian W et al.(2015) | 1.284 | 1.007-1.636 | | |
|  | BBvs. BA+AA | Kim EJ et al.(2005) | 1.221 | 0.986-1.514 | | Random |
|  |  | Garcia-Closas M et al.(2007) | 1.228 | 0.983-1.533 | | |
|  |  | VanCleave TT et al.(2010) | 1.194 | 0.966-1.476 | | |
|  |  | Ajaz S et al.(2011) | 1.194 | 0.964-1.477 | | |
|  |  | Henrquez-Hernndez et al.(2012) | 1.181 | 0.959-1.454 | | |
|  |  | Senz-Lpez P et al. (2013) | 1.192 | 0.956-1.485 | | |
|  |  | Ianni M et al. (2013) | 1.154 | 0.942-1.415 | | |
|  |  | Jaiswal PK et al. (2013) | 1.250 | 1.032-1.515 | | |
|  |  | Martinez-Fierro et al (2013) | 1.232 | 1.040-1.458 | | |
|  |  | Shen BL et al. (2015) | 1.157 | 0.937-1.428 | | |
|  |  | Lu GJ et al. (2015) | 1.157 | 0.932-1.437 | | |
|  |  | Xian W et al. (2015) | 1.154 | 0.936-1.422 | | |
| VEGF-rs833061 | B vs. A | Lin et al. (2003) | 1.030 | 0.942-1.125 | | Random |
|  |  | Fukuda et al. (2007) | 0.985 | 0.876-1.107 | | |
|  |  | Garcia-Closas M et al. (2007) | 0.964 | 1.837-1.109 | | |
|  |  | Onen et al. (2008) | 0.972 | 0.865-1.092 | | |
|  |  | Bruyre F et al. (2010) | 1.032 | 0.944-1.127 | | |
|  |  | Senz-Lpez P et al. (2013) | 0.972 | 0.858-1.100 | | |
|  |  | Wang YH et al. (2013)| | 0.974 | 0.860-1.102 | | |
|  |  | Lu GJ et al. (2015) | 0.957 | 0.859-1.065 | | |
|  | BA vs. AA | Lin et al. (2003) | 1.080 | 0.956-1.219 | | Random |
|  |  | Fukuda et al. (2007) | 0.954 | 0.735-1.240 | | |
|  |  | Garcia-Closas M et al. (2007) | 0.938 | 0.704-1.248 | | |
|  |  | Onen et al. (2008) | 0.923 | 0.726-1.173 | | |
|  |  | Bruyre F et al. (2010) | 0.994 | 0.786-1.256 | | |
|  |  | Senz-Lpez P et al. (2013) | 0.941 | 0.729-1.214 | | |
|  |  | Wang YH et al. (2013)| | 0.931 | 0.703-1.233 | | |
|  |  | Lu GJ et al. (2015) | 0.917 | 0.708-1.187 | | |
|  | BA+BB vs. AA | Lin et al. (2003) | 1.086 | 0.929-1.270 | | Random |
|  |  | Fukuda et al. (2007) | 0.946 | 0.723-1.237 | | |
|  |  | Garcia-Closas M et al. (2007) | 0.922 | 0.687-1.239 | | |
|  |  | Onen et al. (2008) | 0.915 | 0.712-1.175 | | |
|  |  | Bruyre F et al. (2010) | 1.018 | 0.814-1.274 | | |
|  |  | Senz-Lpez P et al. (2013) | 0.929 | 0.714-1.210 | | |
|  |  | Wang YH et al. (2013)| | 0.922 | 0.690-1.233 | | |
|  |  | Lu GJ et al. (2015) | 0.897 | 0.690-1.165 | | |
|  | BB vs. AA | Lin et al. (2003) | 1.117 | 0.952-1.310 | | Fixed |
|  |  | Fukuda et al. (2007) | 1.122 | 0.952-1.322 | | |
|  |  | Garcia-Closas M et al. (2007) | 1.106 | 0.907-1.349 | | |
|  |  | Onen et al. (2008) | 1.106 | 0.941-1.299 | | |
|  |  | Bruyre F et al. (2010) | 1.170 | 0.997-1.374 | | |
|  |  | Senz-Lpez P et al. (2013) | 1.105 | 0.935-1.306 | | |
|  |  | Wang YH et al. (2013)| | 1.127 | 0.950-1.337 | | |
|  |  | Lu GJ et al. (2015) | 1.014 | 0.842-1.220 | | |
|  | BB vs. BA+AA | Lin et al. (2003) | 1.073 | 0.930-1.239 | | Fixed |
|  |  | Fukuda et al. (2007) | 1.082 | 0.934-1.253 | | |
|  |  | Garcia-Closas M et al. (2007) | 1.059 | 0.878-1.277 | | |
|  |  | Onen et al. (2008) | 1.077 | 0.932-1.245 | | |
|  |  | Bruyre F et al. (2010) | 1.126 | 0.975-1.301 | | |
|  |  | Senz-Lpez P et al. (2013) | 1.074 | 0.923-1.249 | | |
|  |  | Wang YH et al. (2013) | 1.088 | 0.935-1.266 | | |
|  |  | Lu GJ et al. (2015) | 1.004 | 0.853-1.182 | | |
| HIF1α-rs11549465 | B vs. A | Clifford SC et al. (2001) | 1.154 | 0.932-1.428 | | Random |
|  |  | Chau CH et al. (2005) | 1.052 | 0.854-1.296 | | |
|  |  | Orr-Urtreger A et al. (2007) | 1.109 | 0.875-1.406 | | |
|  |  | Li H et al. (2007) | 1.121 | 0.870-1.446 | | |
|  |  | Jacobs EJ et al. (2008) | 1.165 | 0.920-1.474 | | |
|  |  | Nadaoka J et al. (2008) | 1.109 | 0.883-1.393 | | |
|  |  | Foley R et al. (2009) | 1.007 | 0.859-1.180 | | |
|  |  | Morris MR et al. (2009) | 1.158 | 0.923-1.453 | | |
|  |  | Li P et al. (2012) | 1.129 | 0.893-1.426 | | |
|  |  | Qin C et al. (2012) | 1.118 | 0.886-1.411 | | |
|  |  | Fraga et al. (2014) | 1.137 | 0.885-1.461 | | |
|  | BA vs. AA | Clifford SC et al. (2001) | 1.147 | 0.916-1.435 | | Random |
|  |  | Chau CH et al. (2005) | 1.061 | 0.856-1.316 | | |
|  |  | Orr-Urtreger A et al. (2007) | 1.147 | 0.898-1.465 | | |
|  |  | Li H et al. (2007) | 1.125 | 0.868-1.457 | | |
|  |  | Jacobs EJ et al. (2008) | 1.167 | 0.905-1.506 | | |
|  |  | Nadaoka J et al. (2008) | 1.125 | 0.889-1.424 | | |
|  |  | Foley R et al. (2009) | 0.997 | 0.874-1.137 | | |
|  |  | Morris MR et al. (2009) | 1.161 | 0.919-1.467 | | |
|  |  | Li P et al. (2012) | 1.147 | 0.902-1.459 | | |
|  |  | Qin C et al. (2012) | 1.125 | 0.885-1.431 | | |
|  |  | Fraga et al. (2014) | 1.141 | 0.883-1.474 | | |
|  | BA+BB vs. AA | Clifford SC et al. (2001) | 1.159 | 0.924-1.454 | | Random |
|  |  | Chau CH et al. (2005) | 1.060 | 0.851-1.320 | | |
|  |  | Orr-Urtreger A et al. (2007) | 1.135 | 0.883-1.459 | | |
|  |  | Li H et al. (2007) | 1.129 | 0.866-1.473 | | |
|  |  | Jacobs EJ et al. (2008) | 1.174 | 0.911-1.514 | | |
|  |  | Nadaoka J et al. (2008) | 1.124 | 0.884-1.429 | | |
|  |  | Foley R et al. (2009) | 1.001 | 0.862-1.163 | | |
|  |  | Morris MR et al. (2009) | 1.168 | 0.920-1.483 | | |
|  |  | Li P et al. (2012) | 1.145 | 0.895-1.465 | | |
|  |  | Qin C et al. (2012) | 1.129 | 0.883-1.442 | | |
|  |  | Fraga et al. (2014) | 1.146 | 0.882-1.489 | | |
|  | BB vs. AA | Clifford SC et al. (2001) | 1.065 | 0.605-1.875 | | Random |
|  |  | Chau CH et al. (2005) | 0.922 | 0.518-1.641 | | |
|  |  | Orr-Urtreger A et al. (2007) | 0.789 | 0.502-1.239 | | |
|  |  | Li H et al. (2007) | 1.064 | 0.535-2.116 | | |
|  |  | Jacobs EJ et al. (2008) | 1.197 | 0.703-2.039 | | |
|  |  | Nadaoka J et al. (2008) | 0.954 | 0.550-1.656 | | |
|  |  | Foley R et al. (2009) | 1.007 | 0.580-1.749 | | |
|  |  | Morris MR et al. (2009) | 1.092 | 0.595-2.002 | | |
|  |  | Li P et al. (2012) | 0.955 | 0.549-1.662 | | |
|  |  | Qin C et al. (2012) | 1.021 | 0.562-1.855 | | |
|  |  | Fraga et al. (2014) | 1.100 | 0.562-2.153 | | |
|  | BB vs. BA+AA | Clifford SC et al. (2001) | 1.052 | 0.605-1.831 | | Random |
|  |  | Chau CH et al. (2005) | 0.926 | 0.524-1.635 | | |
|  |  | Orr-Urtreger A et al. (2007) | 0.770 | 0.507-1.171 | | |
|  |  | Li H et al. (2007) | 1.061 | 0.541-2.080 | | |
|  |  | Jacobs EJ et al. (2008) | 1.182 | 0.699-1.997 | | |
|  |  | Nadaoka J et al. (2008) | 0.947 | 0.553-1.622 | | |
|  |  | Foley R et al. (2009) | 0.998 | 0.582-1.711 | | |
|  |  | Morris MR et al. (2009) | 1.077 | 0.595-1.951 | | |
|  |  | Li P et al. (2012) | 0.948 | 0.552-1.626 | | |
|  |  | Qin C et al. (2012) | 1.013 | 0.565-1.816 | | |
|  |  | Fraga et al. (2014) | 1.092 | 0.566-2.105 | | |
| HIF1α-rs11549467 | B vs. A | Clifford SC et al. (2001) | 1.188 | 0.936-1.508 | | Fixed |
|  |  | Chau CH et al. (2005) | 1.174 | 0.926-1.489 | | |
|  |  | Orr-Urtreger A et al. (2007) | 1.177 | 0.927-1.494 | | |
|  |  | Li H et al. (2007) | 1.220 | 0.949-1.568 | | |
|  |  | Nadaoka J et al. (2008) | 1.253 | 0.965-1.627 | | |
|  |  | Morris MR et al. (2009) | 1.222 | 0.951-1.569 | | |
|  |  | Li P et al. (2012) | 1.017 | 0.768-1.346 | | |
|  |  | Qin C et al. (2012) | 1.188 | 0.896-1.575 | | |
|  | BA vs. AA | Clifford SC et al. (2001) | 1.101 | 0.861-1.409 | | Fixed |
|  |  | Chau CH et al. (2005) | 1.088 | 0.851-1.391 | | |
|  |  | Orr-Urtreger A et al. (2007) | 1.090 | 0.852-1.396 | | |
|  |  | Li H et al. (2007) | 1.123 | 0.866-1.457 | | |
|  |  | Nadaoka J et al. (2008) | 1.200 | 0.918-1.568 | | |
|  |  | Morris MR et al. (2009) | 1.161 | 0.897-1.502 | | |
|  |  | Li P et al. (2012) | 0.921 | 0.688-1.234 | | |
|  |  | Qin C et al. (2012) | 1.064 | 0.793-1.427 | | |
|  | BA+BB vs. AA | Clifford SC et al. (2001) | 1.146 | 0.898-1.463 | | Fixed |
|  |  | Chau CH et al. (2005) | 1.132 | 0.887-1.444 | | |
|  |  | Orr-Urtreger A et al. (2007) | 1.135 | 0.889-1.449 | | |
|  |  | Li H et al. (2007) | 1.174 | 0.907-1.518 | | |
|  |  | Nadaoka J et al. (2008) | 1.229 | 0.942-1.604 | | |
|  |  | Morris MR et al. (2009) | 1.193 | 0.924-1.541 | | |
|  |  | Li P et al. (2012) | 0.968 | 0.725-1.291 | | |
|  |  | Qin C et al. (2012) | 1.126 | 0.843-1.505 | | |
|  | BB vs. AA | Clifford SC et al. (2001) | 5.602 | 0.951-33.009 | | Fixed |
|  |  | Chau CH et al. (2005) | 5.602 | 0.951-33.009 | | |
|  |  | Orr-Urtreger A et al. (2007) | 5.602 | 0.951-33.009 | | |
|  |  | Li H et al. (2007) | 5.602 | 0.951-33.009 | | |
|  |  | Nadaoka J et al. (2008) | 4.050 | 0.449-36.531 | | |
|  |  | Morris MR et al. (2009) | 6.185 | 0.702-54.456 | | |
|  |  | Li P et al. (2012) | 6.872 | 0.796-59.307 | | |
|  |  | Qin C et al. (2012) | 5.602 | 0.951-33.009 | | |
|  | BB vs. BA+AA | Clifford SC et al. (2001) | 5.642 | 0.959-33.209 | | Fixed |
|  |  | Chau CH et al. (2005) | 5.642 | 0.959-33.209 | | |
|  |  | Orr-Urtreger A et al. (2007) | 5.642 | 0.959-33.209 | | |
|  |  | Li H et al. (2007) | 5.642 | 0.959-33.209 | | |
|  |  | Nadaoka J et al. (2008) | 4.039 | 0.449-36.370 | | |
|  |  | Morris MR et al. (2009) | 6.190 | 0.704-54.422 | | |
|  |  | Li P et al. (2012) | 7.029 | 0.814-60.680 | | |
|  |  | Qin C et al. (2012) | 5.642 | 0.959-33.209 | | |
| eNOS-rs1799983 | B vs. A | Medeiros R et al. (2002) | 1.243 | 1.026-1.507 | | Random |
|  |  | Marangoni et al. (2006) | 1.242 | 1.029-1.500 | | |
|  |  | Jacobs EJ et al. (2008) | 1.279 | 1.026-1.594 | | |
|  |  | Lee KM et al. (2009) | 1.277 | 1.024-1.594 | | |
|  |  | Lee KM et al. (2009) | 1.274 | 1.058-1.534 | | |
|  |  | Ryk C et al. (2011) | 1.253 | 1.032-1.522 | | |
|  |  | Ziaei SA et al. (2013) | 1.241 | 1.028-1.499 | | |
|  |  | Safarinejad MR et al. (2013) | 1.248 | 1.030-1.512 | | |
|  |  | Verim L et al. (2013) | 1.208 | 1.005-1.452 | | |
|  |  | Brankovic A et al. (2013) | 1.249 | 1.032-1.511 | | |
|  |  | Polat F et al. (2015) | 1.211 | 1.006-1.457 | | |
|  |  | Ceylan CG et al. (2016) | 1.234 | 1.023-1.488 | | |
|  |  | Diler SB et al. (2016) | 1.059 | 0.965-1.161 | | |
|  | BA vs. AA | Medeiros R et al. (2002) | 1.442 | 1.086-1.914 | | Random |
|  |  | Marangoni et al. (2006) | 1.408 | 1.069-1.855 | | |
|  |  | Jacobs EJ et al. (2008) | 1.540 | 1.094-2.167 | | |
|  |  | Lee KM et al. (2009) | 1.545 | 1.112-2.147 | | |
|  |  | Lee KM et al. (2009) | 1.499 | 1.133-1.983 | | |
|  |  | Ryk C et al. (2011) | 1.489 | 1.117-1.985 | | |
|  |  | Ziaei SA et al. (2013) | 1.494 | 1.133-1.970 | | |
|  |  | Safarinejad MR et al. (2013) | 1.475 | 1.105-1.969 | | |
|  |  | Verim L et al. (2013) | 1.319 | 1.024-1.699 | | |
|  |  | Brankovic A et al. (2013) | 1.461 | 1.100-1.940 | | |
|  |  | Polat F et al. (2015) | 1.300 | 1.015-1.665 | | |
|  |  | Ceylan CG et al. (2016) | 1.464 | 1.108-1.934 | | |
|  |  | Diler SB et al. (2016) | 1.210 | 0.989-1.481 | | |
|  | BA+BB vs. AA | Medeiros R et al. (2002) | 1.449 | 1.098-1.912 | | Random |
|  |  | Marangoni et al. (2006) | 1.424 | 1.086-1.867 | | |
|  |  | Jacobs EJ et al. (2008) | 1.555 | 1.114-2.171 | | |
|  |  | Lee KM et al. (2009) | 1.555 | 1.124-2.152 | | |
|  |  | Lee KM et al. (2009) | 1.507 | 1.147-1.980 | | |
|  |  | Ryk C et al. (2011) | 1.488 | 1.122-1.972 | | |
|  |  | Ziaei SA et al. (2013) | 1.480 | 1.126-1.945 | | |
|  |  | Safarinejad MR et al. (2013) | 1.477 | 1.114-1.957 | | |
|  |  | Verim L et al. (2013) | 1.328 | 1.035-1.704 | | |
|  |  | Brankovic A et al. (2013) | 1.466 | 1.111-1.934 | | |
|  |  | Polat F et al. (2015) | 1.313 | 1.028-1.678 | | |
|  |  | Ceylan CG et al. (2016) | 1.456 | 1.108-1.913 | | |
|  |  | Diler SB et al. (2016) | 1.192 | 0.989-1.435 | | |
|  | BB vs. AA | Medeiros R et al. (2002) | 1.649 | 1.036-2.625 | | Random |
|  |  | Marangoni et al. (2006) | 1.681 | 1.076-2.626 | | |
|  |  | Jacobs EJ et al. (2008) | 1.778 | 1.051-3.009 | | |
|  |  | Lee KM et al. (2009) | 1.749 | 1.007-3.039 | | |
|  |  | Lee KM et al. (2009) | 1.639 | 1.063-2.529 | | |
|  |  | Ryk C et al. (2011) | 1.642 | 1.029-2.621 | | |
|  |  | Ziaei SA et al. (2013) | 1.563 | 0.998-2.447 | | |
|  |  | Safarinejad MR et al. (2013) | 1.603 | 1.030-2.493 | | |
|  |  | Verim L et al. (2013) | 1.498 | 0.970-2.313 | | |
|  |  | Brankovic A et al. (2013) | 1.647 | 1.046-2.594 | | |
|  |  | Polat F et al. (2015) | 1.506 | 0.973-2.333 | | |
|  |  | Ceylan CG et al. (2016) | 1.576 | 1.007-2.467 | | |
|  |  | Diler SB et al. (2016) | 1.118 | 0.868-1.439 | | |
|  | BB vs. BA+AA | Medeiros R et al. (2002) | 1.119 | 0.835-1.498 | | Random |
|  |  | Marangoni et al. (2006) | 1.135 | 0.864-1.491 | | |
|  |  | Jacobs EJ et al. (2008) | 1.203 | 0.889-1.626 | | |
|  |  | Lee KM et al. (2009) | 1.170 | 0.830-1.648 | | |
|  |  | Lee KM et al. (2009) | 1.114 | 0.849-1.462 | | |
|  |  | Ryk C et al. (2011) | 1.092 | 0.818-1.458 | | |
|  |  | Ziaei SA et al. (2013) | 1.053 | 0.809-1.370 | | |
|  |  | Safarinejad MR et al. (2013) | 1.101 | 0.835-1.452 | | |
|  |  | Verim L et al. (2013) | 1.114 | 0.837-1.485 | | |
|  |  | Brankovic A et al. (2013) | 1.114 | 0.839-1.479 | | |
|  |  | Polat F et al. (2015) | 1.133 | 0.850-1.511 | | |
|  |  | Ceylan CG et al. (2016) | 1.075 | 0.818-1.412 | | |
|  |  | Diler SB et al. (2016) | 0.908 | 0.776-1.063 | | |
| eNOS-rs2070744 | B vs. A | Ryk C et al. (2011) | 1.354 | 1.140-1.609 | | Fixed |
|  |  | Safarinejad MR et al. (2013) | 1.326 | 1.108-1.588 | | |
|  |  | Brankovic A et al. (2013) | 1.462 | 1.241-1.724 | | |
|  |  | Polat F et al. (2015) | 1.397 | 1.189-1.641 | | |
|  |  | Diler SB et al. (2016) | 1.349 | 1.149-1.585 | | |
|  | BA vs. AA | Ryk C et al. (2011) | 1.224 | 0.942-1.590 | | Fixed |
|  |  | Safarinejad MR et al. (2013) | 1.077 | 0.830-1.399 | | |
|  |  | Brankovic A et al. (2013) | 1.318 | 1.039-1.673 | | |
|  |  | Polat F et al. (2015) | 1.221 | 0.966-1.544 | | |
|  |  | Diler SB et al. (2016) | 1.295 | 1.026-1.636 | | |
|  | BA+BB vs. AA | Ryk C et al. (2011) | 1.373 | 1.071-1.761 | | Fixed |
|  |  | Safarinejad MR et al. (2013) | 1.246 | 0.973-1.596 | | |
|  |  | Brankovic A et al. (2013) | 1.495 | 1.191-1.877 | | |
|  |  | Polat F et al. (2015) | 1.389 | 1.111-1.735 | | |
|  |  | Diler SB et al. (2016) | 1.411 | 1.129-1.764 | | |
|  | BB vs. AA | Ryk C et al. (2011) | 1.949 | 1.339-2.836 | | Fixed |
|  |  | Safarinejad MR et al. (2013) | 2.014 | 1.344-3.017 | | |
|  |  | Brankovic A et al. (2013) | 2.425 | 1.659-3.545 | | |
|  |  | Polat F et al. (2015) | 2.162 | 1.501-3.115 | | |
|  |  | Diler SB et al. (2016) | 1.962 | 1.352-2.847 | | |
|  | BB vs. BA+AA | Ryk C et al. (2011) | 1.779 | 1.265-2.502 | | Fixed |
|  |  | Safarinejad MR et al. (2013) | 1.975 | 1.358-2.873 | | |
|  |  | Brankovic A et al. (2013) | 2.097 | 1.479-2.974 | | |
|  |  | Polat F et al. (2015) | 1.974 | 1.410-2.765 | | |
|  |  | Diler SB et al. (2016) | 1.702 | 1.207-2.400 | | |
| eNOS-Intron 4a/b VNTR | B vs. A | Mediros R et al. (2002) | 1.366 | 0.910-2.049 | | Random |
|  |  | Sanli O et al. (2011) | 1.530 | 1.072-2.185 | | |
|  |  | Amasyali AS et al. (2012) | 1.276 | 0.892-1.825 | | |
|  |  | Safarinejad MR et al. (2013) | 1.295 | 0.878-1.911 | | |
|  |  | Polat F et al. (2015) | 1.502 | 1.038-2.173 | | |
|  |  | Diler SB et al. (2016) | 1.544 | 1.098-2.172 | | |
|  | BA vs. AA | Mediros R et al. (2002) | 1.291 | 0.815-2.045 | | Random |
|  |  | Sanli O et al. (2011) | 1.415 | 0.912-2.194 | | |
|  |  | Amasyali AS et al. (2012) | 1.165 | 0.878-1.547 | | |
|  |  | Safarinejad MR et al. (2013) | 1.284 | 0.784-2.102 | | |
|  |  | Polat F et al. (2015) | 1.381 | 0.885-2.153 | | |
|  |  | Diler SB et al. (2016) | 1.500 | 1.043-2.158 | | |
|  | BA+BB vs. AA | Mediros R et al. (2002) | 1.376 | 0.855-2.215 | | Random |
|  |  | Sanli O et al. (2011) | 1.535 | 0.992-2.376 | | |
|  |  | Amasyali AS et al. (2012) | 1.241 | 0.880-1.749 | | |
|  |  | Safarinejad MR et al. (2013) | 1.330 | 0.813-2.175 | | |
|  |  | Polat F et al. (2015) | 1.502 | 0.961-2.348 | | |
|  |  | Diler SB et al. (2016) | 1.597 | 1.085-2.351 | | |
|  | BB vs. AA | Mediros R et al. (2002) | 2.640 | 0.923-7.547 | | Random |
|  |  | Sanli O et al. (2011) | 3.615 | 1.545-8.454 | | |
|  |  | Amasyali AS et al. (2012) | 2.375 | 0.813-6.943 | | |
|  |  | Safarinejad MR et al. (2013) | 2.017 | 1.059-3.844 | | |
|  |  | Polat F et al. (2015) | 3.179 | 1.322-7.648 | | |
|  |  | Diler SB et al. (2016) | 2.829 | 1.053-7.602 | | |
|  | BB vs. BA+AA | Mediros R et al. (2002) | 2.762 | 1.563-4.879 | | Fixed |
|  |  | Sanli O et al. (2011) | 3.508 | 1.921-6.406 | | |
|  |  | Amasyali AS et al. (2012) | 2.724 | 1.502-4.941 | | |
|  |  | Safarinejad MR et al. (2013) | 1.737 | 0.935-3.227 | | |
|  |  | Polat F et al. (2015) | 3.013 | 1.731-5.243 | | |
|  |  | Diler SB et al. (2016) | 2.790 | 1.607-4.846 | | |
| HRAS-rs12628 | B vs. A | Johne A et al. (2003) | 1.527 | 0.712-3.271 | | Random |
|  |  | Sanyal S et al. (2004) | 1.679 | 0.956-2.948 | | |
|  |  | Traczyk M et al. (2012) | 1.325 | 0.706-2.484 | | |
|  |  | Pandith AA et al. (2013) | 1.085 | 0.762-1.545 | | |
|  | BA vs. AA | Johne A et al. (2003) | 1.431 | 0.759-2.699 | | Random |
|  |  | Sanyal S et al. (2004) | 1.367 | 0.658-2.843 | | |
|  |  | Traczyk M et al. (2012) | 1.137 | 0.595-2.173 | | |
|  |  | Pandith AA et al. (2013) | 0.926 | 0.655-1.309 | | |
|  | BA+BB vs. AA | Johne A et al. (2003) | 1.552 | 0.708-3.404 | | Random |
|  |  | Sanyal S et al. (2004) | 1.600 | 0.770-3.324 | | |
|  |  | Traczyk M et al. (2012) | 1.243 | 0.617-2.505 | | |
|  |  | Pandith AA et al. (2013) | 1.012 | 0.688-1.487 | | |
|  | BB vs. AA | Johne A et al. (2003) | 1.690 | 0.125-22.768 | | Random |
|  |  | Sanyal S et al. (2004) | 2.537 | 1.135-5.672 | | |
|  |  | Traczyk M et al. (2012) | 1.366 | 0.136-13.740 | | |
|  |  | Pandith AA et al. (2013) | 1.048 | 0.291-3.770 | | |
|  | BB vs. BA+AA | Johne A et al. (2003) | 1.484 | 0.134-16.399 | | Random |
|  |  | Sanyal S et al. (2004) | 2.284 | 1.296-4.024 | | |
|  |  | Traczyk M et al. (2012) | 1.392 | 0.146-13.304 | | |
|  |  | Pandith AA et al. (2013) | 1.082 | 0.331-3.537 | | |

**Supplementary Table 5.** *P* values of the Egger’ test

| **Polymorphism** | **Subgroup** | **Egger’s test *P* > |t|** |
| --- | --- | --- |
| VEGF-rs1570360 | Overall | 0.330 |
|  | PCa | 0.473 |
|  | BCa | 0.359 |
|  | H-B | 0.579 |
|  | P-B | 0.746 |
|  | HWE (Y) | 0.531 |
| VEGF-rs2010963 | Overall | 0.645 |
|  | RCC | 0.705 |
|  | Asian | 0.163 |
|  | Caucasian | 0.249 |
|  | H-B | 0.799 |
|  | HWE (Y) | 0.645 |
| VEGF-rs3025039 | Overall | 0.641 |
|  | RCC | 0.533 |
|  | BCa | 0.302 |
|  | Asian | 0.503 |
|  | Caucasian | 0.136 |
|  | HWE (Y) | 0.409 |
|  | HWE (N) | 0.382 |
| VEGF-rs699947 | Overall | 0.946 |
|  | RCC | 0.520 |
|  | Asian | 0.615 |
|  | Caucasian | 0.915 |
|  | PCa | 0.371 |
|  | BCa | 0.430 |
|  | H-B | 0.896 |
|  | HWE (N) | 0.806 |
| VEGF-rs833061 | Overall | 0.073 |
|  | PCa | 0.463 |
|  | RCC | 0.058 |
|  | Asian | 0.125 |
|  | Caucasian | 0.395 |
|  | H-B | 0.221 |
|  | P-B | 0.374 |
| VEGF-rs10434 | Overall | 0.373 |
| eNOS-rs1799983 | Overall | 0.060 |
|  | PCa | 0.179 |
|  | BCa | 0.159 |
|  | H-B | 0.421 |
|  | P-B | 0.228 |
| eNOS-rs2070744 | Overall | 0.516 |
|  | PCa | 0.804 |
|  | H-B | 0.903 |
| eNOS-Intron 4a/b VNTR | Overall | 0.071 |
|  | H-B | 0.473 |
|  | PCa | 0.267 |
| HIF1α-rs11549465 | Overall | 0.229 |
|  | PCa | 0.061 |
|  | RCC | 0.442 |
|  | Asian | 0.275 |
|  | Caucasian | 0.756 |
|  | H-B | 0.425 |
|  | HWE (Y) | 0.352 |
|  | HWE (N) | 0.793 |
| HIF1α-rs11549467 | Overall | 0.773 |
|  | Asian | 0.568 |
|  | Caucasian | 0.735 |
|  | H-B | 0.607 |
|  | HWE (Y) | 0.456 |
| HRAS-rs12628 | Overall | 0.318 |
|  | Caucasian | 0.868 |

**Supplementary Table 6.** Summary of LD analyses

| VEGF | CEU | # | Name | Position | ObsHET | PredHET | HWpval | %Geno | FamTrio | MendErr | MAF | Alleles | Rating |
| --- | --- | --- | --- | --- | --- | --- | --- | --- | --- | --- | --- | --- | --- |
|  |  | 1 | rs699947 | 43736389 | 0.505 | 0.496 | 1 | 100 | 0 | 0 | 0.455 | C : A |  |
|  |  | 2 | rs833061 | 43737486 | 0.495 | 0.495 | 1 | 100 | 0 | 0 | 0.449 | T : C |  |
|  |  | 3 | rs1570360 | 43737830 | 0.384 | 0.406 | 0.7237 | 100 | 0 | 0 | 0.283 | G : A |  |
|  |  | 4 | rs2010963 | 43738350 | 0.455 | 0.441 | 0.9861 | 100 | 0 | 0 | 0.328 | G : C |  |
|  |  | 5 | rs3025039 | 43752536 | 0.263 | 0.257 | 1 | 100 | 0 | 0 | 0.152 | C : T |  |
|  |  | 6 | rs10434 | 43753212 | 0.515 | 0.487 | 0.7501 | 100 | 0 | 0 | 0.419 | G : A |  |
|  | CHB | # | Name | Position | ObsHET | PredHET | HWpval | %Geno | FamTrio | MendErr | MAF | Alleles | Rating |
|  |  | 1 | rs699947 | 43736389 | 0.427 | 0.396 | 0.6229 | 100 | 0 | 0 | 0.272 | C : A |  |
|  |  | 2 | rs833061 | 43737486 | 0.427 | 0.396 | 0.6229 | 100 | 0 | 0 | 0.272 | T : C |  |
|  |  | 3 | rs1570360 | 43737830 | 0.33 | 0.288 | 0.2724 | 100 | 0 | 0 | 0.175 | G : A |  |
|  |  | 4 | rs2010963 | 43738350 | 0.602 | 0.494 | 0.0484 | 100 | 0 | 0 | 0.447 | G : C |  |
|  |  | 5 | rs3025039 | 43752536 | 0.291 | 0.301 | 0.9263 | 100 | 0 | 0 | 0.184 | C : T |  |
|  |  | 6 | rs10434 | 43753212 | 0.359 | 0.33 | 0.6059 | 100 | 0 | 0 | 0.209 | G : A |  |
|  | JPT | # | Name | Position | ObsHET | PredHET | HWpval | %Geno | FamTrio | MendErr | MAF | Alleles | Rating |
|  |  | 1 | rs699947 | 43736389 | 0.442 | 0.447 | 1 | 100 | 0 | 0 | 0.337 | C : A |  |
|  |  | 2 | rs833061 | 43737486 | 0.442 | 0.447 | 1 | 100 | 0 | 0 | 0.337 | T : C |  |
|  |  | 3 | rs1570360 | 43737830 | 0.298 | 0.28 | 0.8331 | 100 | 0 | 0 | 0.168 | G : A |  |
|  |  | 4 | rs2010963 | 43738350 | 0.5 | 0.473 | 0.7532 | 100 | 0 | 0 | 0.385 | G : C |  |
|  |  | 5 | rs3025039 | 43752536 | 0.24 | 0.267 | 0.4627 | 100 | 0 | 0 | 0.159 | C : T |  |
|  |  | 6 | rs10434 | 43753212 | 0.269 | 0.233 | 0.2443 | 100 | 0 | 0 | 0.135 | G : A |  |
|  | YRI | # | Name | Position | ObsHET | PredHET | HWpval | %Geno | FamTrio | MendErr | MAF | Alleles | Rating |
|  |  | 1 | rs699947 | 43736389 | 0.222 | 0.212 | 1 | 100 | 0 | 0 | 0.12 | C : A |  |
|  |  | 2 | rs833061 | 43737486 | 0.463 | 0.417 | 0.3866 | 100 | 0 | 0 | 0.296 | T : C |  |
|  |  | 3 | rs1570360 | 43737830 | 0.065 | 0.063 | 1 | 100 | 0 | 0 | 0.032 | G : A |  |
|  |  | 4 | rs2010963 | 43738350 | 0.444 | 0.424 | 0.8373 | 100 | 0 | 0 | 0.306 | G : C |  |
|  |  | 5 | rs3025039 | 43752536 | 0.139 | 0.129 | 1 | 100 | 0 | 0 | 0.069 | C : T |  |
|  |  | 6 | rs10434 | 43753212 | 0.398 | 0.38 | 0.8521 | 100 | 0 | 0 | 0.255 | G : A |  |
| eNOS | CEU | # | Name | Position | ObsHET | PredHET | HWpval | %Geno | FamTrio | MendErr | MAF | Alleles | Rating |
|  |  | 1 | rs2070744 | 150690079 | 0.566 | 0.491 | 0.2099 | 100 | 0 | 0 | 0.434 | T : C |  |
|  |  | 2 | rs1799983 | 150696111 | 0.525 | 0.463 | 0.2804 | 100 | 0 | 0 | 0.364 | G : T |  |
|  | CHB | # | Name | Position | ObsHET | PredHET | HWpval | %Geno | FamTrio | MendErr | MAF | Alleles | Rating |
|  |  | 1 | rs2070744 | 150690079 | 0.223 | 0.198 | 0.502 | 100 | 0 | 0 | 0.112 | T : C |  |
|  |  | 2 | rs1799983 | 150696111 | 0.252 | 0.235 | 0.832 | 100 | 0 | 0 | 0.136 | G : T |  |
|  | JPT | # | Name | Position | ObsHET | PredHET | HWpval | %Geno | FamTrio | MendErr | MAF | Alleles | Rating |
|  |  | 1 | rs2070744 | 150690079 | 0.202 | 0.182 | 0.6511 | 100 | 0 | 0 | 0.101 | T : C |  |
|  |  | 2 | rs1799983 | 150696111 | 0.125 | 0.15 | 0.2587 | 100 | 0 | 0 | 0.082 | G : T |  |
|  | YRI | # | Name | Position | ObsHET | PredHET | HWpval | %Geno | FamTrio | MendErr | MAF | Alleles | Rating |
|  |  | 1 | rs2070744 | 150690079 | 0.25 | 0.219 | 0.3119 | 100 | 0 | 0 | 0.125 | T : C |  |
|  |  | 2 | rs1799983 | 150696111 | 0.111 | 0.105 | 1 | 100 | 0 | 0 | 0.056 | G : T |  |
| HIF-1α | CEU | # | Name | Position | ObsHET | PredHET | HWpval | %Geno | FamTrio | MendErr | MAF | Alleles | Rating |
|  |  | 1 | rs11549465 | 62207557 | 0.131 | 0.123 | 1 | 100 | 0 | 0 | 0.066 | C : T |  |
|  |  | 2 | rs11549467 | 62207575 | 0.01 | 0.01 | 1 | 100 | 0 | 0 | 0.005 | G : A |  |
|  | CHB | # | Name | Position | ObsHET | PredHET | HWpval | %Geno | FamTrio | MendErr | MAF | Alleles | Rating |
|  |  | 1 | rs11549465 | 62207557 | 0.068 | 0.084 | 0.3334 | 100 | 0 | 0 | 0.044 | C : T |  |
|  |  | 2 | rs11549467 | 62207575 | 0.058 | 0.057 | 1 | 100 | 0 | 0 | 0.029 | G : A |  |
|  | JPT | # | Name | Position | ObsHET | PredHET | HWpval | %Geno | FamTrio | MendErr | MAF | Alleles | Rating |
|  |  | 1 | rs11549465 | 62207557 | 0.077 | 0.074 | 1 | 100 | 0 | 0 | 0.038 | C : T |  |
|  |  | 2 | rs11549467 | 62207575 | 0.058 | 0.056 | 1 | 100 | 0 | 0 | 0.029 | G : A |  |
|  | YRI | # | Name | Position | ObsHET | PredHET | HWpval | %Geno | FamTrio | MendErr | MAF | Alleles | Rating |
|  |  | 1 | rs11549465 | 62207557 | 0.056 | 0.054 | 1 | 100 | 0 | 0 | 0.028 | C : T |  |
|  |  | 2 | rs11549467 | 62207575 | 0 | 0 | 1 | 100 | 0 | 0 | 0 | G : G | BAD |

ObsHET: observed height; PredHET: Predicted height; HWpval: Hardy-Weinberg equilibrium p value; FamTrio: the number of fully genotyped family trios for this marker; MendErr: the number of observed Mendelian inheritance errors; MAF: Minor allele frequency; CEU: Utah residents with Northern and Western European ancestry from the CEPH collection; CHB: Han Chinese in Beijing, China; JPT: Japanese in Tokyo, Japan; YRI: Yoruba in Ibadan, Nigeria.
